# Design of Differential Single‐Block and Multi‐Block Presaturated Ultrashort Echo Time Pulse Sequences for Fast and Flexible Short‐T_2_ Imaging

**DOI:** 10.1002/mrm.70379

**Published:** 2026-04-20

**Authors:** Jason A. Reich, Shannon L. Taylor, Kevin D. Harkins, Rachelle L. Crescenzi, Erin L. MacMillan, Rebecca E. Feldman

**Affiliations:** ^1^ Department of Computer Science, Mathematics, Physics and Statistics University of British Columbia Kelowna British Columbia Canada; ^2^ Department of Biomedical Engineering Vanderbilt University Nashville Tennessee USA; ^3^ Vanderbilt University Institute of Imaging Science Vanderbilt University Medical Center Nashville Tennessee USA; ^4^ Department of Radiology and Radiological Sciences Vanderbilt University Medical Center Nashville Tennessee USA; ^5^ UBC MRI Research Centre, Department of Radiology University of British Columbia Vancouver British Columbia Canada; ^6^ Djavad Mowafaghian Centre for Brain Health University of British Columbia Vancouver British Columbia Canada; ^7^ Biomedical Engineering and Imaging Institute Icahn School of Medicine at Mount Sinai New York New York USA

**Keywords:** human, pulse sequence design, reduced field‐of‐view, RF pulse design, short‐T_2_ imaging, simultaneous multi‐slice, skull

## Abstract

**Purpose:**

Ultrashort echo time (UTE) techniques are essential for the magnetic resonance imaging of short‐*T*_2_ signals. Current UTE techniques are time‐consuming and often require the acquisition of an inflexible volume. In this work, we implement new UTE sequences—differential single‐block presaturated UTE (δSB‐UTE) and differential multi‐block presaturated UTE (δMB‐UTE)—that combine spatial presaturation and subtraction to enable simultaneous multi‐slice UTE acquisition with increased flexibility in field‐of‐view (FOV) and reduced scan times.

**Methods:**

The sequences use a prepulse to presaturate blocks of magnetization and a whole‐volume excitation to enable UTE acquisition. By shifting the saturation blocks and subtracting subsequent acquisitions, two and four simultaneous slices can be imaged with the δSB‐UTE and δMB‐UTE sequences, respectively. Design trade‐offs were investigated in a phantom, and the sequences were used to image healthy volunteers at 3 T.

**Results:**

The received signal strength increased as prepulse flip angle and saturation block shift increased. Slice thickness increased as prepulse flip angle, saturation block shift, and saturation block thickness decreased, and prepulse time·bandwidth product increased. The δSB‐UTE and δMB‐UTE sequences were used to image a 210 × 210 × 80 mm^3^ in vivo volume with 1.0 × 1.0 × 4.0 mm^3^ resolution in 2.66 and 1.45 min, respectively.

**Conclusion:**

The δSB‐UTE and δMB‐UTE sequences can be used to capture short‐T_2_ signal from the skull in multiple slices at the same time with an echo time of 0.18 ms. The δSB‐UTE and δMB‐UTE sequences enable the acquisition of a reduced through‐plane FOV and reduce scan times for imaging bone.

## 1 Introduction

Tissues that contain spin populations with short nuclear magnetic resonance transverse relaxation times (*T*_2_ = 0.26–1.8 ms), such as tendons [1, 2, 3, 4], ligaments [3, 4], and bone [5, 6, 7, 8], are challenging to detect with the echo times (TEs) used in most magnetic resonance imaging (MRI) techniques. Routinely implemented spin echo and gradient echo sequences require radiofrequency (RF) or gradient refocussing that limits minimum TEs to a few milliseconds [9]. With long TEs, transverse magnetization dephases before signal acquisition begins, causing tissues that contain short‐T_2_ spin populations to appear dark in resulting images. To produce images that contain as much short‐T_2_ signal as possible, ultrashort echo time (UTE) pulse sequences with TEs of 8–200 μs have been developed [1, 2, 3, 4, 5, 6, 7, 8, 10, 11, 12, 13, 14, 15, 16, 17].

Three‐dimensional (3D) UTE sequences commonly use a non‐selective rectangular RF pulse to excite the entire anatomy within the transmit volume of the RF coils [10, 11, 12, 13, 18]. Non‐selective pulses do not require refocusing and allow data acquisition to begin once the RF coils switch from transmit to receive. Data is acquired with a centre‐out trajectory to fill a 3D k‐space. The use of a 3D acquisition [19] often limits the minimum FOV in at least one direction, resulting in scan times of 8.2–15.3 min for 0.7–1.6 mm isotropic resolutions [10, 11, 12, 13].

Two‐dimensional (2D) UTE sequences enable the manual selection of the location and number of slices. Slice‐selective excitation that enables UTE acquisition can be performed with half RF pulses [14, 15, 16, 17, 20, 21] and the TE can be shortened using the variable rate selective excitation (VERSE) algorithm [22] to make use of the ramp‐down time of the slice select gradient. Like non‐selective pulses, half pulses do not require refocusing and data acquisition begins once the RF coils switch from transmit to receive. However, a second repetition using a slice‐select gradient with opposing polarity is necessary as each half pulse only partially excites the slice. Data is acquired with a centre‐out trajectory to fill a 2D k‐space slice‐by‐slice. Half‐pulse UTE sequences have required total scan times of 4.4–6.6 min per slice (including both half‐excitations) for 0.4–0.9 mm in‐plane resolutions [14, 15, 16, 17] and have been shown to be susceptible to gradient errors that lead to slice profile imperfections [23, 24, 25].

The saturation‐based UTE (sat‐UTE) [26] sequence, which combines spatial presaturation and subtraction, can be used to perform 2D short‐T_2_ imaging that is robust to gradient imperfections. Spatial presaturation is a strategy also used for reduced field‐of‐view (FOV) imaging [27] and it is often combined with subtraction in arterial spin labelling [28]. With the sat‐UTE sequence, a non‐selective pulse is used for excitation and slice selection is performed by subtracting 2D acquisitions with and without a saturated slice.

Simultaneous multi‐slice (SMS) techniques [29, 30, 31] have reduced scan times for applications in anatomical [32], functional [33], diffusion‐weighted [34], and perfusion‐weighted [35] imaging. The simultaneous excitation of multiple slices can be achieved by superimposing single‐slice pulses [29] or by using power independent of number of slices (PINS) [36] pulses. Signal can be localized by leveraging spatial information from a multi‐channel RF coil with sensitivity encoding (SENSE) [37, 38]. Signal localization can be improved by performing phase encoding along the slice‐select direction with controlled aliasing in parallel imaging (CAIPI) [39, 40]. For radial acquisitions, CAIPI enables the estimation coil sensitivity maps directly from the acquired data [41, 42], eliminating the need for a reference scan and further reducing scan times.

To date, SMS and 2D UTE techniques have been challenging to combine. One approach has involved the use of a PINS prepulse to saturate long‐T_2_ signals so that superimposed half‐pulses can be used to excite the remaining short‐T_2_ signals in four simultaneous slices [43]. Another approach has involved the use of a PINS prepulse to saturate wide regions of unwanted magnetization so that a non‐selective whole‐volume excitation can be used to excite three simultaneous slices [44, 45]. With both approaches, image quality has been degraded by out‐of‐slice signal and scan times have been limited by safety limits on power deposition.

In this work, we design and implement differential single‐block presaturated UTE (δSB‐UTE) and differential multi‐block presaturated UTE (δMB‐UTE) sequences [46] to increase flexibility in FOV and reduce scan times for short‐T_2_ imaging. The δSB‐UTE and δMB‐UTE sequences use spatial presaturation and subtraction to integrate SMS techniques into UTE sequences. We investigate design trade‐offs in a phantom and use the sequences to image short‐T_2_ signals from in vivo human skulls.

## 2 Methods

### 2.1 Pulse Sequence Design

**FIGURE 1:**
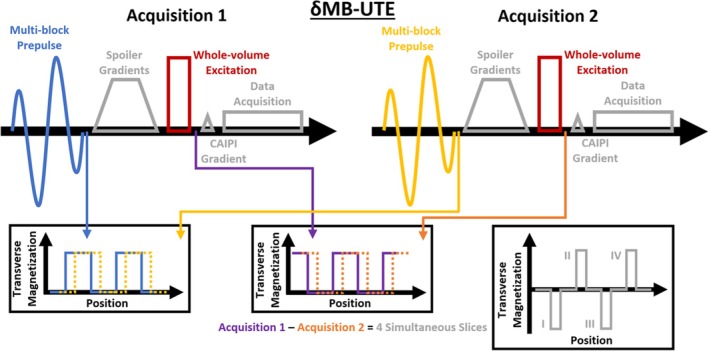
A schematic diagram of the δSB‐UTE sequence showing transverse magnetization throughout the sequence and the subtraction of subsequent acquisitions. In the first acquisition, a multi‐block prepulse (blue) tips wide regions of magnetization into the transverse plane (blue) and spoiler gradients dephase the transverse magnetization to create saturation blocks. A non‐selective, rectangular pulse (red) excites the magnetization surrounding the saturation blocks (purple) and data acquisitions begins following the CAIPI gradient and transmit/receive switching. In the second acquisition, frequency modulation of the prepulse (yellow) shifts the saturation blocks (orange) along the slice‐select direction. Subtraction of subsequent acquisitions enables the reconstruction of four simultaneous slices (I, II, III, and IV).

The δSB‐UTE and δMB‐UTE sequences use spatial presaturation and subtraction to image short‐T_2_ signals with SMS techniques. The δSB‐UTE and δMB‐UTE sequences begin with a prepulse to tip one or two blocks of magnetization, respectively, into the transverse plane, leaving the surrounding magnetization along the longitudinal axis. Spoiler gradients dephase the transverse magnetization and a whole‐volume excitation excites the surrounding magnetization. The δMB‐UTE sequence is illustrated in Figure 1 (see Figure S1 for the δSB‐UTE sequence). Data acquisition with a centre‐out 2D k‐space trajectory begins once the RF coils switch from transmit to receive. By subtracting subsequent acquisitions with saturation blocks shifted along the slice‐select direction, two slices can be obtained simultaneously with the δSB‐UTE sequence, and four slices can be obtained simultaneously with the δMB‐UTE sequence. The simultaneous slices are disentangled with SENSE and CAIPI. To enable CAIPI, a short gradient is applied along the slice select direction while the RF coils are switching from transmit to receive.

The δSB‐UTE and δMB‐UTE sequences reported here were designed to meet the hardware specifications of a 3 T Philips Ingenia CX, Ingenia Elition X, and MR7700 (Philips, Best, The Netherlands) equipped with a 32‐channel receive‐only head coil. Prepulses with flip angles (*α*_pre_) of 30°–120° were designed in MATLAB R2023b (MathWorks, Natick, Massachusetts, United States of America) using the VERSE algorithm in the Multiband RF Toolbox (https://github.com/mriphysics/Multiband‐RF) [47]. For the δSB‐UTE sequence, prepulses with time·bandwidth products (TBWs) of 6.6, 13.2, and 19.8 and slice thicknesses of 20, 40, and 60 mm (698–1306 μs durations) were designed to saturate a single block of magnetization. For the δMB‐UTE sequence, prepulses with TBWs of 3.3, 6.6, and 9.9 and slice thicknesses of 10, 20, and 30 mm (774–1651 μs durations) were designed to saturate two blocks of magnetization with a centre‐to‐centre separation equal to twice the slice thickness. Spoiler gradients were designed with areas of 23.5 ms·mT/m (to cause 2·π dephasing over 1 mm) and applied on all axes. For whole‐volume excitation, non‐selective pulses with Ernst flip angles [48] (*α*_ex_ = 24.9°–51.5° and 75–154 μs durations) were used. The CAIPI gradient had a duration of 0.089 and 0.125 ms for the δSB‐UTE and δMB‐UTE sequences, respectively. Additional details about sequence design and specific absorption rate (SAR) calculation can be found in the Supporting Information (Sections S1 and S2, respectively).

### 2.2 Simulations

Simulations were performed in Python 3.12 (Python Software Foundation, Wilmington, DE, United States of America) using a Bloch simulator (https://github.com/namalkanti/bloch‐simulator‐python). Transverse magnetization profiles were simulated with *α*_pre_ = 70°, a single spoiler gradient on the through‐plane axis, *α*_ex_ = 70°, and no CAIPI gradient. The δSB‐UTE sequence was simulated with a prepulse TBW of 13.2 and saturation block thickness of 40 mm. The δMB‐UTE sequence was simulated with a prepulse TBW of 6.6 and saturation block thickness of 20 mm. Two sets of steady‐state simulations were performed using: (1) repetition time (TR) = 35 ms, *α*_ex_ = 29.9°, *T*_2_ = 0.1–10 ms, and longitudinal relaxation time (*T*_1_) = 245 ms [49, 50, 51]; (2) TR = 30–150 ms, *α*_ex_ = 27.8°–57.2°, *T*_2_ = 5 ms, and *T*_1_ = 245 ms. Additionally, longitudinal magnetization profiles after a single repetition of each 70° prepulse were simulated using *T*_1_ = 245 ms and *T*_2_ = 5 ms under two conditions: (1) frequency offsets of −800 to 800 Hz and no B_0_ scaling; (2) B_0_ scale factors of 0.4–1.6 and no frequency offset. A 150 mm through‐plane FOV was simulated with 15 000 points that were binned in groups of 86, emulating a 0.86 mm resolution. Slice profiles were estimated by subtracting the transverse magnetization profiles with a saturation block shift of 4 mm and taking the absolute value.

Slice profile characteristics were analyzed for each slice. The profile maximum was determined by taking the maximum value and the full width at half maximum (FWHM) was calculated using linear interpolation. The total signal was determined by integrating over 10 mm in each direction from the profile maximum. The location of the slice was assessed by taking the profile‐weighted average over the regions that were integrated to determine total signal. The shift away from the centre of the profile was determined by taking the difference between the simulated and expected slice positions.

### 2.3 MRI Experiments

All experiments were performed on the 3 T scanners listed in Section 2.1. Four healthy human volunteers (females aged 38, 42, and 52 years, male aged 25 years) were imaged with Institutional Review Board approval after obtaining written, informed consent.

Slice profiles were investigated in a periodic image quality test (PIQT) phantom (Philips, Best, The Netherlands) by acquiring slice and saturation block projections (images of signal projected onto the coronal plane) without the CAIPI gradient. Data acquisition was performed using a single‐echo radial acquisition. For Ernst angle calculation, *T*_1_ = 317 ms and all TEs are reported from the centre of the excitation. Unless otherwise stated, parameters included: *α*_pre_ = 70°, a saturation block shift of 4 mm, TR = 53 ms, *α*_ex_ = 32.2°, TE = 0.18 ms, number of readouts (*N*_ro_) = 424, number of readout samples (*N*_s_) = 253, resolution = 1.0 × 1.0 mm^2^, and FOV = 210 × 210 mm^2^; The δSB‐UTE sequence used a prepulse TBW of 13.2 and saturation block thickness of 40 mm; The δMB‐UTE sequence used a prepulse TBW of 6.6 and saturation block thickness of 20 mm. The following four experiments were performed: (1) The δSB‐UTE and δMB‐UTE sequences were constructed with *α*_pre_ = 30°–120° and TR = 150 and 53 ms. For TR = 150 ms, *α*_ex_ = 51.5° and TE = 0.21 ms. For TR = 53 ms, *α*_ex_ = 32.2° and TE = 0.18 ms. Additional projections were obtained with a TR = 33 ms, *α*_pre_ = 70°, *α*_ex_ = 25.7°, and TE = 0.18 ms. (2) The δSB‐UTE sequence was created with saturation block thicknesses of 20, 40, and 60 mm; the δMB‐UTE sequence was created with saturation block thicknesses of 10, 20, and 30 mm. (3) The δSB‐UTE sequence was implemented with prepulse TBWs of 6.6, 13.2, and 19.8; the δMB‐UTE sequence was implemented with prepulse TBWs of 3.3, 6.6, and 13.2. (4) Each sequence was executed with saturation block shifts of 1–10 mm. The parameters used for slice profile investigation are summarized in Table S1. Images and corresponding slice profiles were obtained in the PIQT phantom as described in the Supporting Information (Section S3).

All in vivo experiments were performed using a dual‐echo radial acquisition with TR = 34 ms, *α*_ex_ = 29.5° (Ernst angle for *T*_1_ = 245 ms [49, 50, 51]), and TE_1_/TE_2_ = 0.18/2.34 ms. The following three experiments were performed: (1) Axial images were obtained with 2 acquisitions, *N*_ro_ = 400, *N*_s_ = 253/400 (TE_1_/TE_2_), resolution = 1.0 × 1.0 mm^2^, and FOV = 200 × 200 mm^2^. Slice projections corresponding to the axial images were obtained with *N*_ro_ = 360, *N*_s_ = 216/360 (TE_1_/TE_2_), resolution = 1.0 × 1.0 mm^2^, and FOV = 180 × 180 mm^2^. (2) Sagittal images were obtained to demonstrate the imaging of an 80 mm through‐plane FOV with *N*_ro_ = 424, *N*_s_ = 234/424 (TE_1_/TE_2_), resolution = 1.0 × 1.0 mm^2^, and FOV = 210 × 210 mm^2^. To image 20 slices, the δSB‐UTE sequence required 11 acquisitions, resulting in a scan time of 2.66 min, while the δMB‐UTE sequence required 6 acquisitions, resulting in a scan time of 1.45 min. (3) Sagittal images were obtained to compare the δSB‐UTE and δMB‐UTE sequences to a 3D UTE sequence with matched voxel volume and scan time. The 3D UTE sequence used a kooshball trajectory [10, 11, 12, 18] with *N*_ro_ = 29 540, *N*_s_ = 163/280 (TE_1_/TE_2_), resolution = 1.57 mm (isotropic), FOV = 220 mm (isotropic), and scan time = 16.75 min. The δSB‐UTE and δMB‐UTE sequences used 2 acquisitions, *N*_ro_ = 448, *N*_s_ = 247/448 (TE_1_/TE_2_), resolution = 0.98 × 0.98 × 4 mm^3^, and FOV = 220 × 220 mm^2^. For the δSB‐UTE and δMB‐UTE sequences 6 and 11 scan averages were obtained, respectively, so that the time required to image an 80 mm through‐plane FOV would be 16.77 min. The remaining parameters for all in vivo experiments were the same as for phantom slice profile experiment 1 (see Table S3). Additional motion tolerance experiments were performed as described in the Supporting Information (Section S4).

### 2.4 Reconstruction and Analysis

Image reconstruction was performed offline using ReconFrame (Gyrotools LLC, Zurich, Switzerland) and the Berkeley Advanced Reconstruction Toolbox (BART, https://mrirecon.github.io/bart/) [52] in MATLAB R2023b. Data was corrected with the “BasicCorrections” method and sorted with the “SortData” method in ReconFrame. K‐space trajectories were estimated with the “traj” command in BART and the trajectory of echo 1 was modified to account for ramp sampling.

Slice and saturation block projections were reconstructed using the “nufft” command in BART. Projections obtained from the PIQT phantom and projections obtained in vivo were reconstructed with in‐plane resolutions of 0.73 × 0.73 and 0.70 × 0.70 mm^2^, respectively. Slice profiles were calculated from the slice projections by averaging 20 lines perpendicular to the excited slices in a region where the slices were uniform. Slice profiles were normalized by the average signal from regions of interest (ROIs) outside of the saturation blocks. Profile maximum, total signal, and FWHM were determined as described in Section 2.2, except slice profiles were integrated over a symmetric region with a width that is 1.5 times the distance between the outer edges of the saturation blocks (containing all of the slices). Each measure was normalized by the number of slices.

Before reconstructing images, readout lines were partitioned based on their CAIPI gradient encoding and phase was corrected according to the position of the slices. Coil sensitivity maps were estimated using the “nlinv” command in BART with 20 iterations and a calibration region that included only data in the centre of k‐space satisfying the Nyquist criterion. Joint echo reconstruction was performed using the “pics” command in BART with ℓ_1_‐wavelet regularization, a regularization strength of 0.0001, and 100 iterations. Sagittal in vivo images were reconstructed with an in‐plane resolution of 0.73 × 0.73 mm^2^. Axial in vivo images were reconstructed with an in‐plane resolution of 0.69 × 0.69 mm^2^. Normalized difference images were calculated by subtracting echo 2 from echo 1 and dividing by echo 1. Images from echo 1 and echo 2 were scaled by subtracting the mean and dividing by the standard deviation within a region masked to exclude the background and were displayed showing pixel values from the 1st to 99th percentile.

For in vivo experiment 3, images were reconstructed with an in‐plane resolution of 0.69 × 0.69 mm^2^ using the method described for in vivo experiments 1 and 2 without regularization. For the data obtained with the 3D UTE sequence, coil sensitivity maps were estimated using only half of the data satisfying the Nyquist criterion and 200 iterations. Images obtained with the 3D UTE sequence were reconstructed with a through‐plane resolution of 4.00 mm (to match the slice thickness of the δSB‐UTE and δMB‐UTE sequences) using 1000 iterations. Contrast‐to‐noise ratio (CNR) was calculated as the difference in signal‐to‐noise ratio (SNR) between regions of interest within the skull and brain tissue. SNR was calculated as the mean magnitude within each ROI divided by the standard deviation of the real component of an image obtained with no RF or gradients. Edge strength at the boundary between the skull and brain tissue was also calculated as described previously [53]. Slice leakage and noise amplification were evaluated as described in the Supporting Information (Section S5).

## 3 Results

### 3.1 Simulations

**FIGURE 2:**
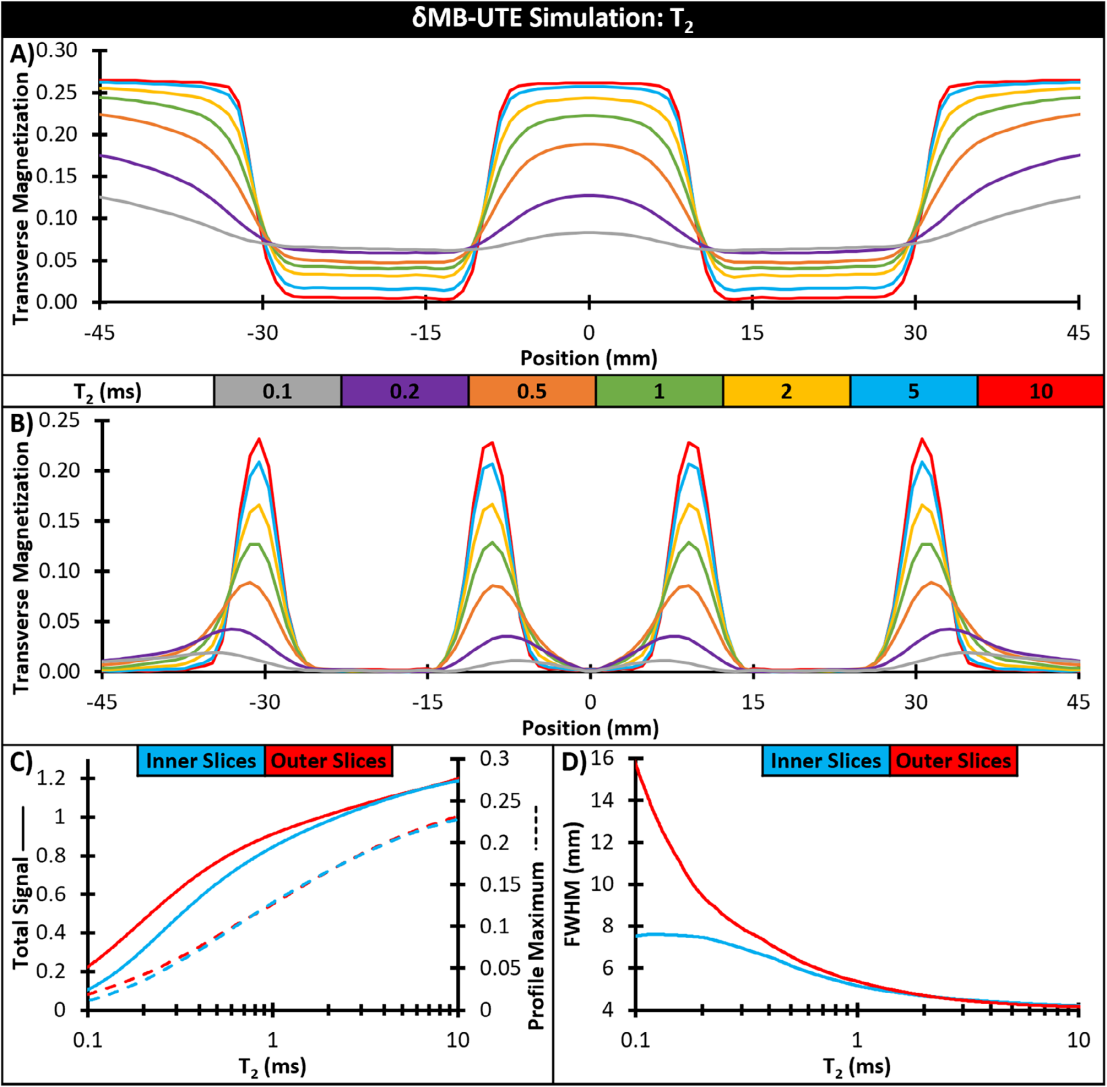
Slice profiles of δMB‐UTE sequence simulated with *T*_2_ = 0.1–10 ms. Parameters included a prepulse flip angle of 70°, prepulse TBW of 6.6, saturation block thickness of 20 mm, TR of 35 ms, excitation flip angle of 29.9°, and T_1_ of 245 ms. The saturation block was not shifted to produce saturation block profiles (A) but was shifted 4 mm to produce slice profiles (B). The total signal and profile maximum (C), and FWHM (D) are plotted for each slice.

The steady‐state saturation block and slice profiles of the δMB‐UTE sequence simulated with *T*_2_ = 0.1–10 ms are plotted in Figure 2A,B, respectively. Figure 2C shows the total signal and profile maximum of the inner and outer slices, revealing a decrease in signal within the inner slices for *T*_2_ < 1 ms as a result of the broadening of saturation blocks. Figure 2D shows the FWHM for the inner and outer slices and reveals increased short‐T_2_ broadening of the outer slices. For *T*_2_ > 1 ms, profile maxima are greater than half that of *T*_2_ = 10 ms and the FWHM is < 5.4 mm. The profiles of the δSB‐UTE sequence were similar to those of the outer slices of the δMB‐UTE sequence (see Figure S3). The RF and gradient waveforms of the prepulses used for simulation can be found in Figure S2, and the remaining simulation results can be found in Figures S4–S7.

### 3.2 Phantom Experiments

**FIGURE 3:**
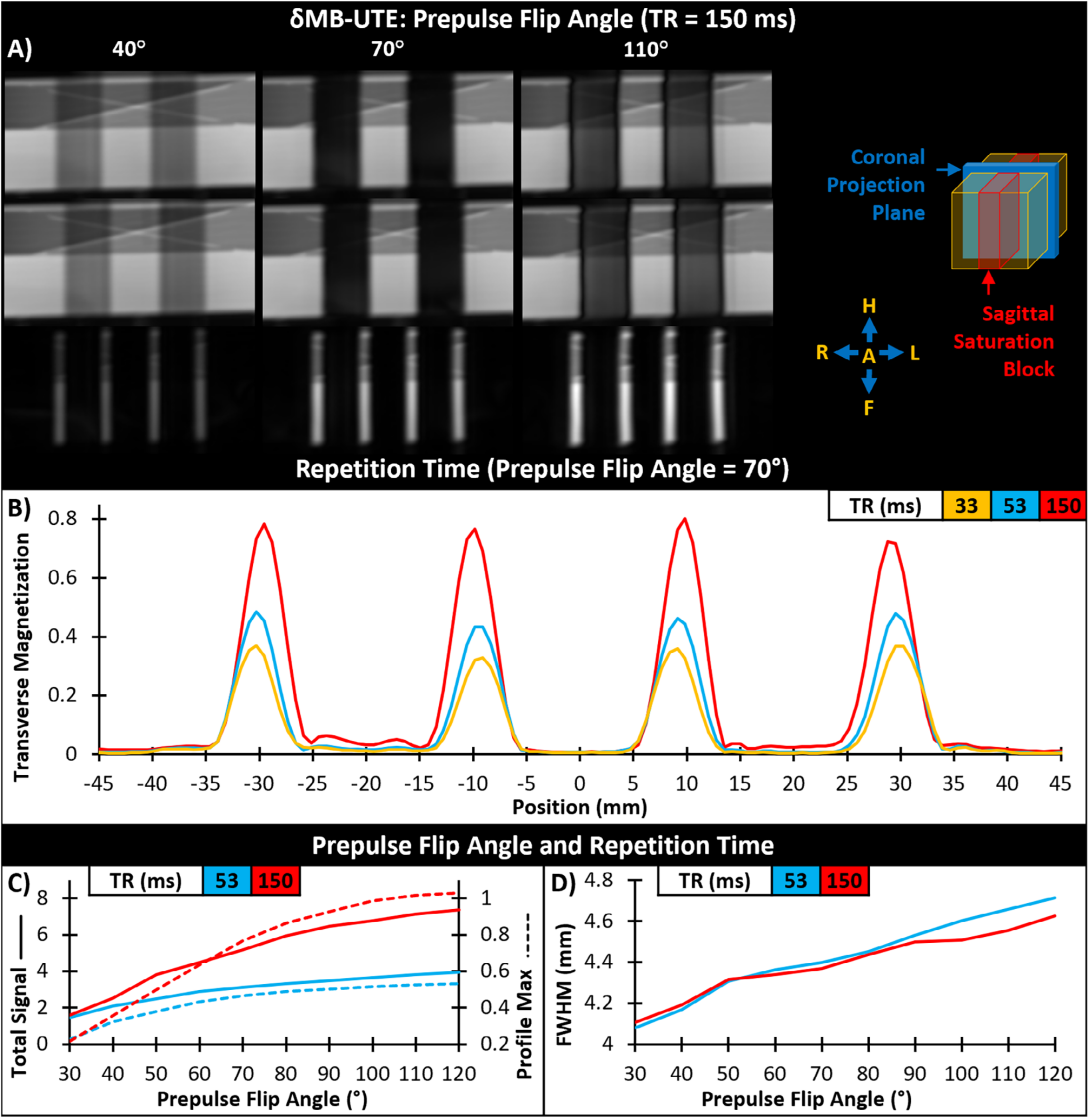
Results of varying prepulse flip angle and TR (phantom experiment 1) for the δMB‐UTE sequence. Saturation block and slice projections obtained with prepulse flip angles of 40°, 70°, and 110° and a TR of 150 ms (A). Slice profiles obtained using TRs of 33, 53, and 150 ms and a prepulse flip angle of 70° (B). Plots of total signal (solid) and profile maximum (dashed) (C), and FWHM (D) for prepulse flip angles of 30°–120° and TRs of 53 (blue) and 150 (red) ms. Other parameters included a saturation block thickness of 20 mm, prepulse TBW of 6.6, saturation block shift of 4 mm, and excitation flip angles of 25.7°–51.5°.

The results of varying *α*_pre_ and TR (phantom experiment 1) for the δMB‐UTE sequence are illustrated in Figure 3. Saturation block and slice projections (Figure 3A) and plots of total signal and profile maximum (Figure 3C) show that signal within the slices increases with *α*_pre_ as a result of an increased difference between magnetization within and surrounding the saturation block. Increasing *α*_pre_ up to 70° results in improved saturation, while increasing *α*_pre_ further inverts magnetization within the saturation block, causing it to be excited out of phase with the surrounding magnetization. SAR also increases with *α*_pre_, limiting the minimum TR (see Table S1). Slice profiles (Figure 3B) illustrate that total signal and profile maximum are reduced and become less dependent on *α*_pre_ for shorter TRs. In addition, slices shift away from the saturation blocks as TR is shortened, in agreement with simulations (Figure S3). The FWHM is plotted in Figure 3D and increases with *α*_pre_ as the transition width between saturated and excited magnetization increases. To achieve reasonable SNR with the shortest possible scan time, *α*_pre_ = 70° and the minimum TR allowed by SAR limits were used for imaging. Similar results were found for the δSB‐UTE sequence (Figure S8).

Results from the remaining phantom experiments illustrate the trade‐offs between profile maximum, total signal, and FWHM as saturation block thickness, prepulse TBW, and saturation block shift are varied (Figures S9–S14). Decreasing prepulse TBW results in a decrease in profile maximum and an increase in FWHM due to an increase in transition width between saturated and excited magnetization. As saturation block thickness is increased, the saturation block profile is stretched over a larger distance, resulting in an increase in slice separation and FWHM. Increasing saturation block shift results in an increase in FWHM and total signal. Profile maximum also increases with saturation block shift up to 6 mm, but remains constant for larger shifts as transition regions no longer overlap. Images and slice projections obtained in the PIQT phantom, shown in Figures S15 and S16, demonstrate the ability of the δSB‐UTE and δMB‐UTE sequences to rapidly image an 80 mm through‐plane FOV in 2.42 and 1.33 min, respectively.

### 3.3 In Vivo Experiments

**FIGURE 4:**
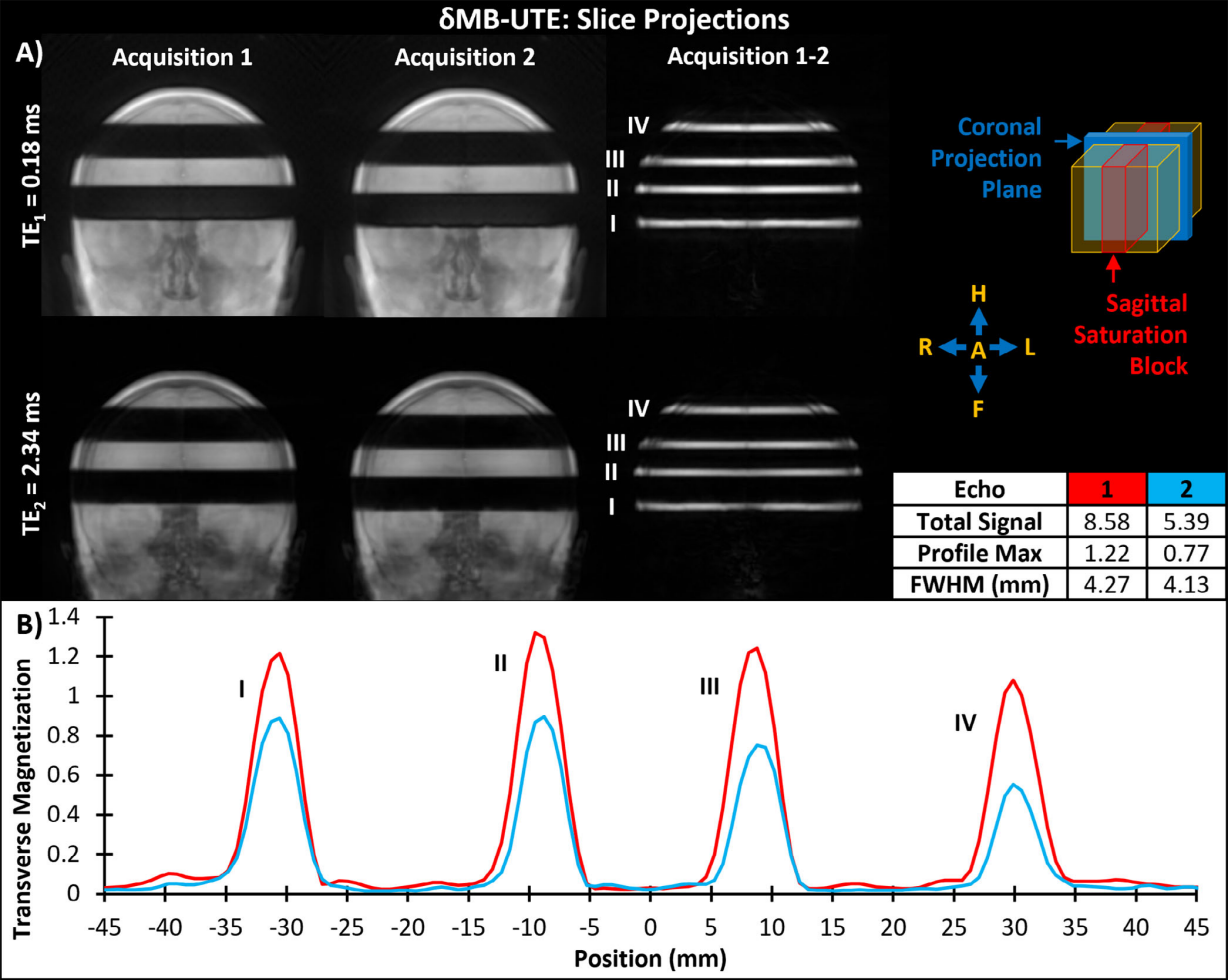
Slice projections (A) and profiles (B) obtained using the δMB‐UTE sequence with TE_1_ = 0.18 ms and TE_2_ = 2.34 ms (in vivo experiment 1). Slices I, II, III, and IV correspond to the axial images in Figure 6. Parameters included a prepulse flip angle of 70°, prepulse TBW of 6.6, saturation block thickness of 20 mm, saturation block shift of 4 mm, and TR of 34 ms, excitation flip angle of 29.5°.

**FIGURE 5:**
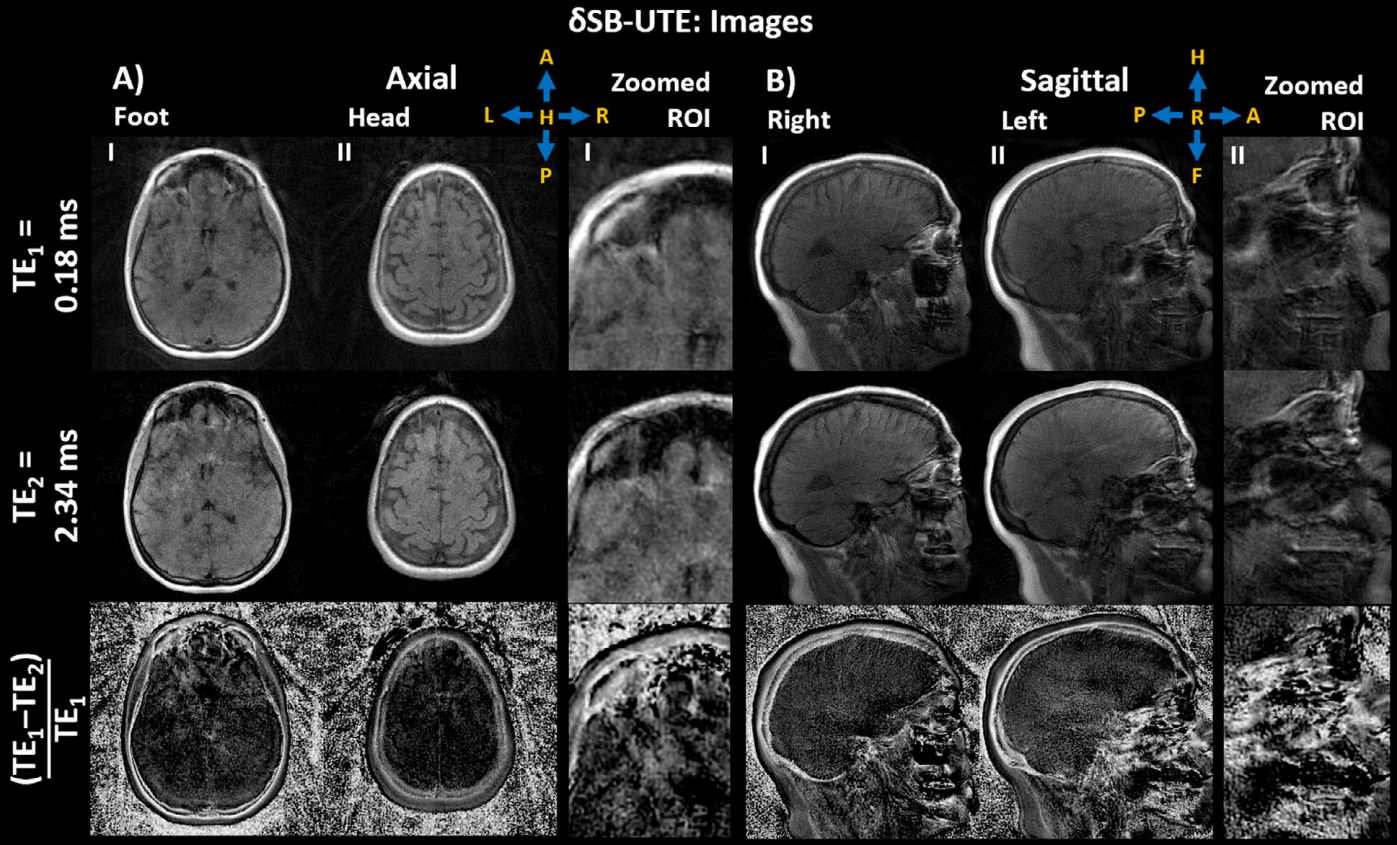
Axial (A) and sagittal (B) images obtained with the δSB‐UTE sequence (in vivo experiments 1 and 2). Images in sets I and II were obtained simultaneously. The top row shows echo 1 (TE_1_ = 0.18 ms), the middle row shows echo 2 (TE_2_ = 2.34 ms), and the bottom row shows the normalized difference, highlighting short‐T_2_ signal from the skull. The rightmost column of each panel shows zoomed ROIs (from slice I for panel A and slice II for panel B) of the oral cavity and orbits, where motion artifacts can be seen. Parameters included a prepulse flip angle of 70°, prepulse TBW of 13.2, saturation block thickness of 40 mm, saturation block shift of 4 mm, TR of 34 ms, and excitation flip angle of 29.5°.

**FIGURE 6:**
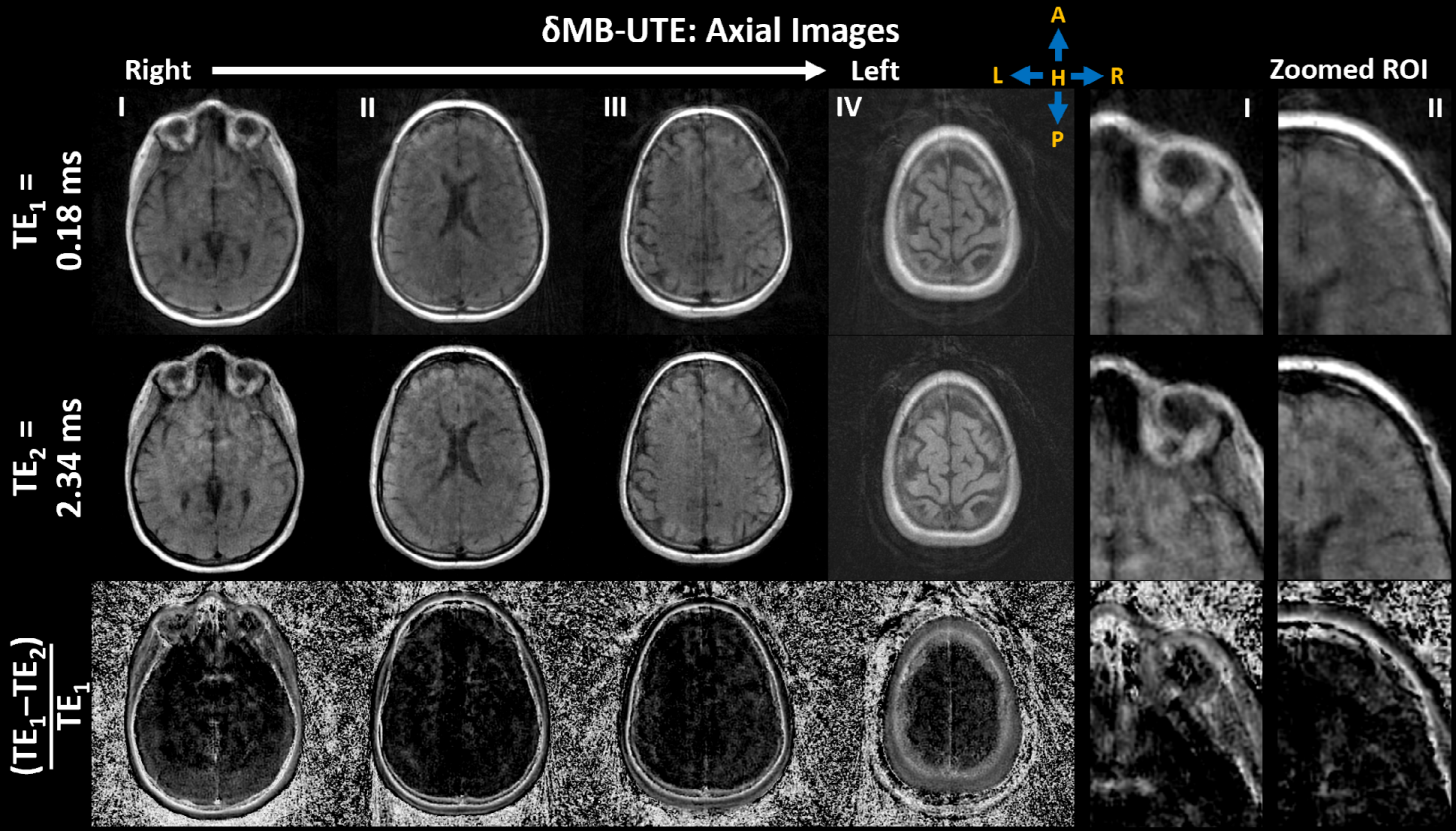
Axial images obtained with the δMB‐UTE sequence (in vivo experiment 1). Images in sets I, II, III, and IV were obtained simultaneously. The top row shows echo 1 (TE1 = 0.18 ms), the middle row shows echo 2 (TE2 = 2.34 ms), and the bottom row shows the normalized difference, highlighting short‐T_2_ signal from the skull. The rightmost columns show zoomed ROIs (from slice I and II) of the oral cavity and orbits, where motion artifacts can be seen. Parameters included a prepulse flip angle of 70°, prepulse TBW of 6.6, saturation block thickness of 20 mm, saturation block shift of 4 mm, TR of 34 ms, and excitation flip angle of 29.5°.

**FIGURE 7:**
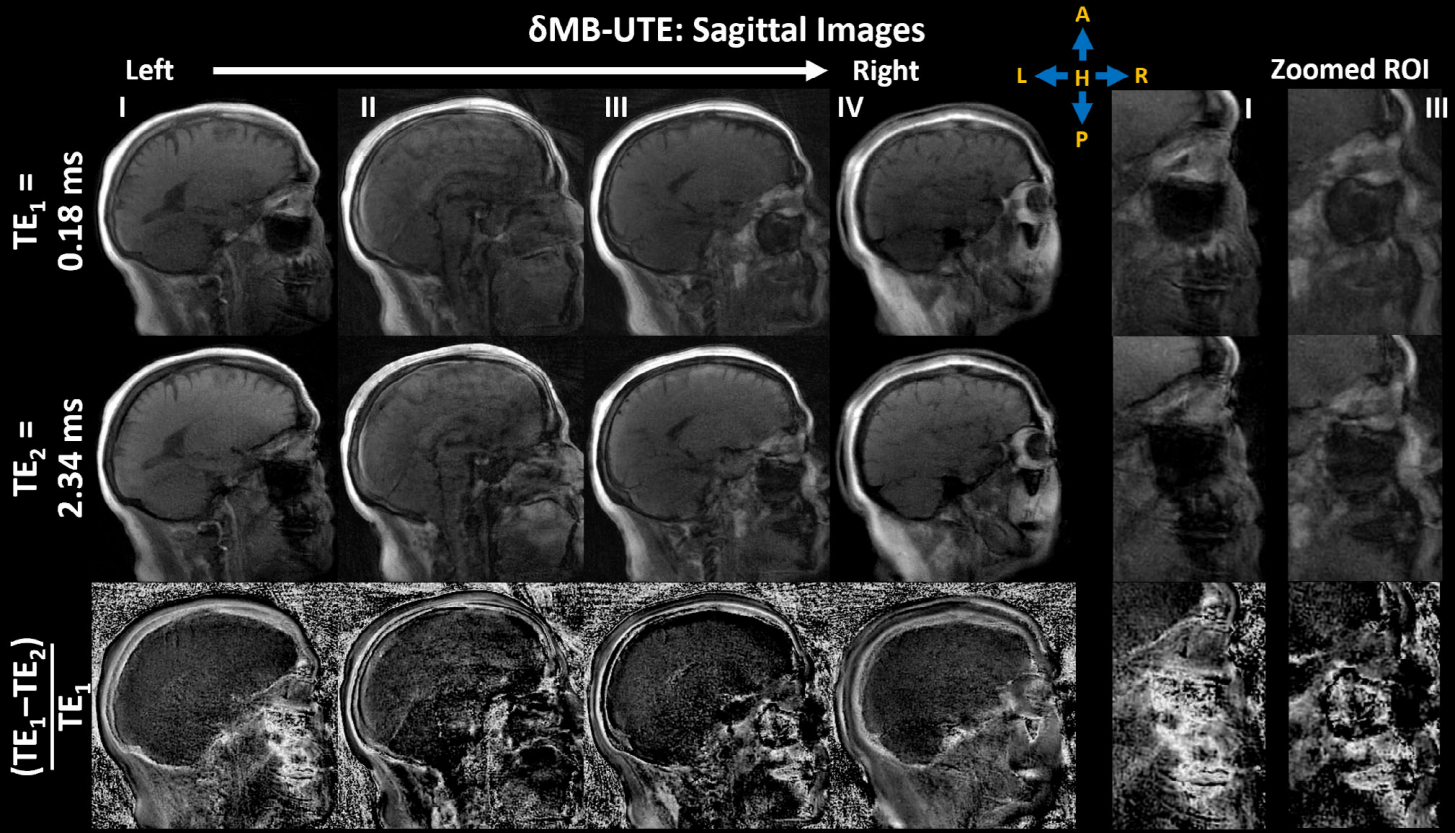
Sagittal images obtained with the δMB‐UTE sequence (in vivo experiment 2). Images in columns I, II, III, and IV were obtained simultaneously. The top row shows echo 1 (TE_1_ = 0.18 ms) and the middle row shows echo 2 (TE_2_ = 2.34 ms). The bottom row shows the normalized difference, highlighting short‐T_2_ signal from the skull. The rightmost columns show zoomed ROIs (from slice I and III) of the oral cavity and orbits, where motion artifacts can be seen. Parameters included a prepulse flip angle of 70°, prepulse TBW of 6.6, saturation block thickness of 20 mm, saturation block shift of 4 mm, TR of 34 ms, and excitation flip angle of 29.5°.

**FIGURE 8:**
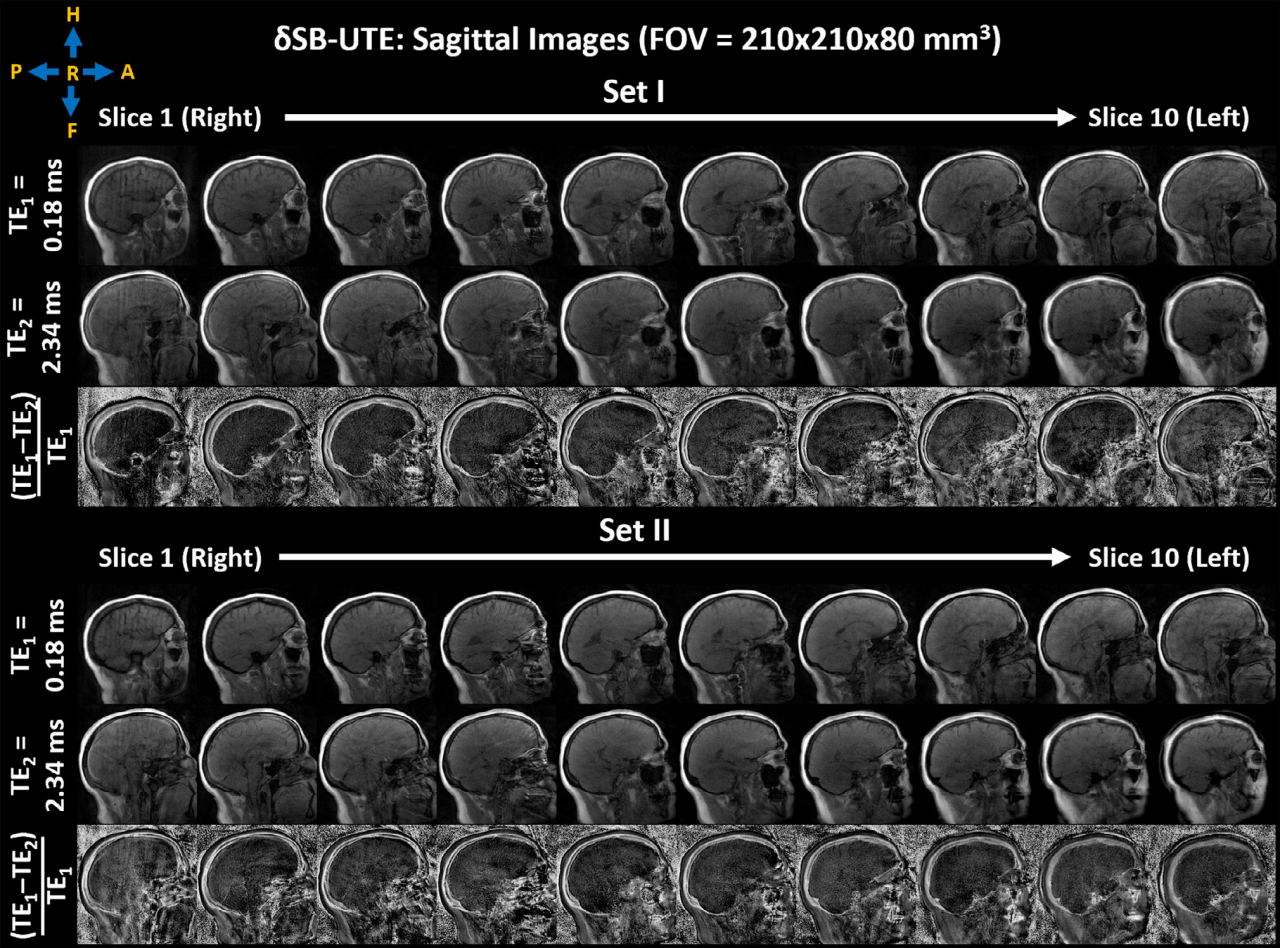
Sagittal images obtained with the δSB‐UTE sequence from an 80 mm through‐plane FOV in 2.66 min (in vivo experiment 2). Images in sets I and II were obtained simultaneously. For each set, the top row shows echo 1 (TE_1_ = 0.18 ms), the middle row shows echo 2 (TE_2_ = 2.34 ms), and the bottom row shows the normalized difference. Parameters were the same as for the acquisition of the sagittal images in Figure 5B.

**FIGURE 9:**
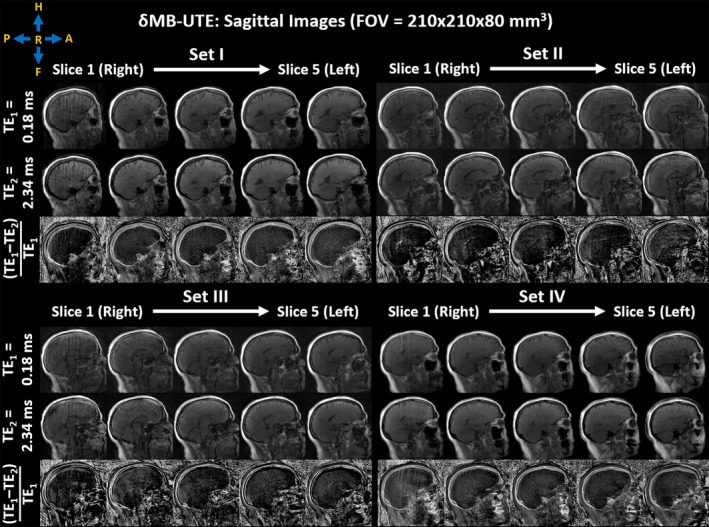
Sagittal images obtained with the δMB‐UTE sequence from an 80 mm through‐plane FOV in 1.45 min (in vivo experiment 2). Images in sets I, II, III, and IV were obtained simultaneously. For each set, the top row shows echo 1 (TE_1_ = 0.18 ms), the middle row shows echo 2 (TE_2_ = 2.34 ms), and the bottom row shows the normalized difference. Parameters were the same as for the acquisition of the sagittal images in Figure 7.

In vivo slice profiles obtained with the δMB‐UTE sequence (in vivo experiment 1) are shown in Figure 4 and demonstrate minimal out‐of‐slice signal. Similar slice profiles were obtained with the δSB‐UTE sequence (Figure S17). The corresponding axial images obtained with the δSB‐UTE sequence and δMB‐UTE sequences are shown in Figures 5A and 6, respectively. Figures 5B and 7 show sagittal images that were obtained simultaneously with each sequence (in vivo experiment 2). The zoomed ROIs in Figures 5, 6, 7 show the orbits and oral cavity in selected slices where motion artifacts are suspected. Images show delineation of gyri and sulci, ventricles, and adipose tissue surrounding the skull. Through‐plane aliasing appears as a faint outline of other simultaneous slices, likely as a result of slices shifting away from the centre of the saturation blocks at short TRs. The remaining sagittal images obtained over the 210 × 210 × 80 mm^3^ FOV using the δSB‐UTE and δMB‐UTE sequences (in vivo experiment 2) are shown in Figures 8 and 9, respectively. These figures demonstrate the image quality that can be achieved when imaging an 80 mm through‐plane FOV. Additional images from motion tolerance experiments can be found in Figures S19–S21. Minimal slice leakage, and noise suppression can be seen in L‐factor and g‐factor maps (Figures S22 and S23, respectively).

**FIGURE 10:**
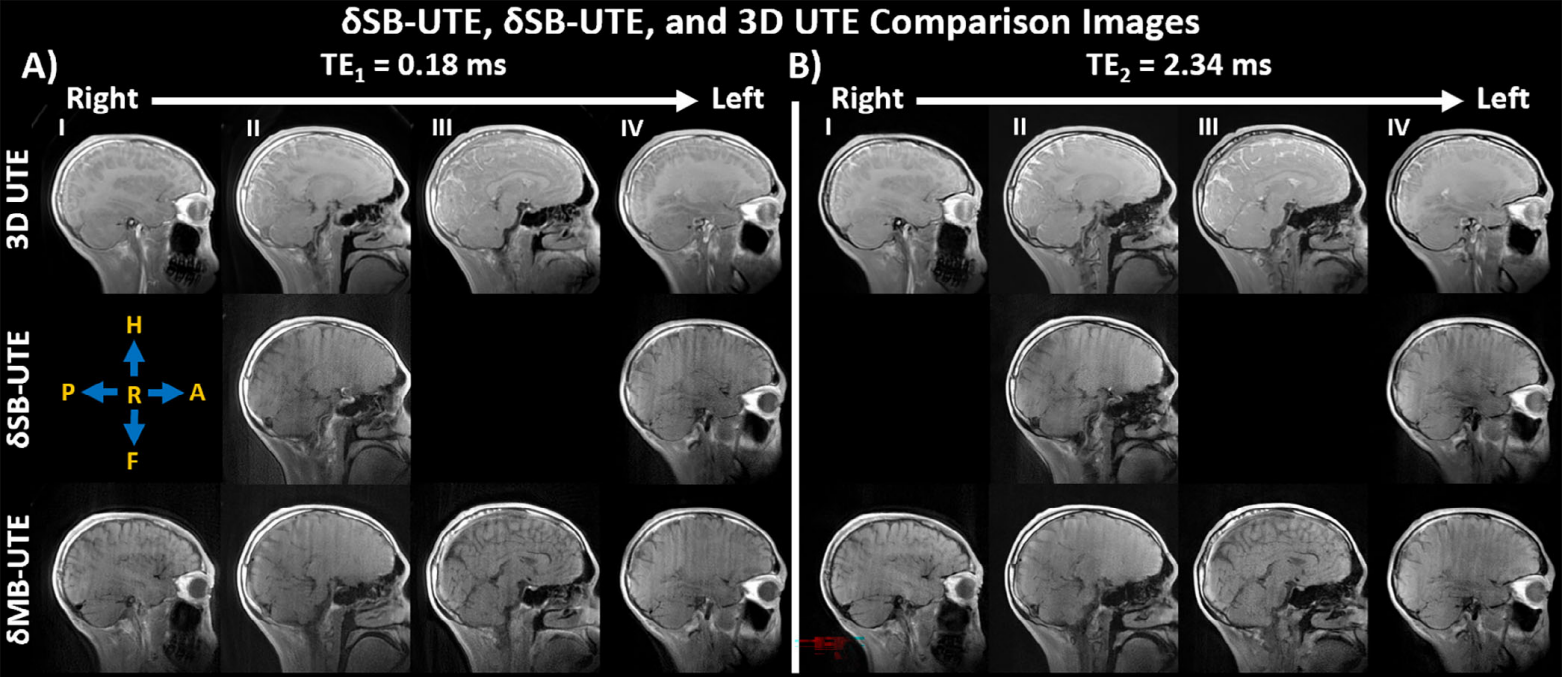
Sagittal images from echo 1 (A) and echo 2 (B) obtained with 3D UTE, δSB‐UTE, and δMB‐UTE sequences (in vivo experiment 3). All images were obtained with a TR of 34 ms, excitation flip angle of 29.5°, voxel volume of 3.87 mm^3^, and in‐plane FOV of 220 × 220 mm^2^, and reconstructed with a resolution of 0.69 × 0.69 × 4 mm^3^. Scan times were matched to ˜16.8 min by acquiring 6 and 11 scan averages with the δSB‐UTE and δMB‐UTE sequences, respectively. The remaining parameters of the δSB‐UTE and δMB‐UTE sequences were the same as for the acquisition of sagittal images in Figures 5B and 7, respectively.

Images obtained with 3D UTE, δSB‐UTE, and δMB‐UTE under matched voxel volumes and scan times (in vivo experiment 3) are shown in Figure 10. In the images of the 3D UTE sequence, CSF appears bright due to incomplete dephasing of magnetization at the end of each repetition, while with the δSB and δMB‐UTE sequences, the spoiler gradients ensure sufficient dephasing. The relative mean of each slice, SNR of bone, CNR between bone and brain tissue, and edge sharpness are tabulated in Table S5. The relative mean of each slice illustrates a reduced signal within the central slices of the δMB‐UTE sequence, in agreement with simulations of short‐T_2_ components (Figures 2 and S2). Images obtained with the δSB‐UTE and δMB‐UTE sequences demonstrate a decrease in SNR and CNR as compared to the 3D UTE sequence, likely due to the use of fewer readout lines per image set and the use of subtraction. Edge sharpness is comparable between the sequences with some variation depending on slice and location.

## 4 Discussion

Using the δSB‐UTE and δMB‐UTE sequences, in vivo images can be obtained from two and four simultaneous slices, respectively, with a TE of 0.18 ms. These sequences can be used to increase flexibility in FOV and reduce scan times for short‐T_2_ imaging.

### 4.1 Comparison to Current Techniques

The δSB‐UTE and δMB‐UTE sequences enable the rapid imaging of short‐T_2_ signals over a reduced through‐plane FOV that is 2 and 4 times the saturation block thickness, respectively. As implemented in this work, a 210 × 210 × 80 mm^3^ FOV can be imaged with 1.0 × 1.0 × 4.0 mm^3^ resolution (4 mm^3^ voxel volume) in 2.66 min using the δSB‐UTE sequence and in 1.45 min using the δMB‐UTE sequence. A 3D UTE sequence with a kooshball acquisition [10, 11, 12, 18] would require 6.77 min to image a 210 mm isotropic FOV with 1.59 mm isotropic resolution (4 mm^3^ voxel volume) and a TR of 5 ms. To achieve comparable contrast, the 3D UTE sequence implemented in this work used a TR of 34 ms that was matched to that of the δSB‐UTE and δMB‐UTE sequences. The resulting scan time of the 3D UTE sequence was 16.75 min, while the δSB‐UTE and δMB‐UTE sequences could be used to image an 80 mm through‐plane FOV with a single average, and the same voxel volume and FOV in 1.53 and 2.80 min, respectively. Although alternate k‐space trajectories [19] and undersampling techniques [37, 54] may accelerate 3D UTE acquisitions, the δMB‐UTE sequence may also be accelerated with modification to acquire more simultaneous slices.

When image quality is limited by SNR, 3D UTE sequences may perform better than the δSB‐UTE and δMB‐UTE sequences. Other 2D acquisitions can be used to achieve SNR efficiencies comparable to 3D acquisitions by interleaving the location of excited slices, thereby increasing the effective TR for each slice [55]. Interleaving excitations cannot be used to improve the SNR of the δSB‐UTE and δMB‐UTE sequences because the whole volume of magnetization is dephased during each repetition. Further, the use of subtraction imposes an SNR penalty of $1/\sqrt{2}$ on the δSB‐UTE and δMB‐UTE sequences due to addition of noise in quadrature.

Identical acquisition parameters can be used to more directly compare the δSB‐UTE and δMB‐UTE sequences to other 2D UTE sequences. A scan time reduction arises from the imaging of N slices with N/2 + 1 and N/4 + 1 acquisitions when using the δSB‐UTE and δMB‐UTE sequences, respectively. The scan time reduction of the δSB‐UTE and δMB‐UTE sequences as compared to other 2D UTE sequences depends on the acquired FOV, with larger through‐plane FOVs resulting in a greater benefit for the δSB‐UTE and δMB‐UTE sequences. For the following calculations, the TR, number of scan averages, resolution, and 210 × 210 × 80 mm^3^ FOV were held constant between the sequences.

Compared to half‐pulse UTE sequences [14, 15, 16, 17, 20, 21], the scan times of the δSB‐UTE and δMB‐UTE sequences are reduced by factors of 3.64 and 6.67, respectively. Each sequence faces an SNR penalty of $1/\sqrt{2}$ from the combination of 2 acquisitions, resulting in SNR efficiencies for the δSB‐UTE and δMB‐UTE sequences that are expected be 1.91 and 2.58 times that of a half‐pulse UTE sequence, respectively.

The sat‐UTE sequence [26] faces the same SNR penalty from subtraction as the δSB‐UTE and δMB‐UTE sequences. Given the same set of parameters listed above, the required scan time of the δSB‐UTE sequence is reduced by a factor of 1.91 and the SNR efficiency is expected to be increased by a factor of 1.38 compared to the sat‐UTE sequence. For the δMB‐UTE sequence, the required scan time is 3.50 times less and the SNR efficiency is expected to be 1.87 times greater than the sat‐UTE sequence. In addition, short‐T_2_‐related slice profile broadening is less than that of the sat‐UTE and half‐pulse UTE sequences [26].

### 4.2 Limitations

The δSB‐UTE and δMB‐UTE sequences are limited by increased motion sensitivity as motion between successive acquisitions hampers the cancellation of signal by subtraction. The result is out‐of‐slice signal that can be seen in phantom slice profiles (particularly in Figures S11, S13, and S14) and image artifacts around the orbits and oral cavity (Figures 5, 6, 7). As motion increases beyond small eye and mouth movements, image quality degrades (Figures S19–21 and Tables S6 and S7). Motion sensitivity may be reduced by acquiring each saturation block shift prior to incrementing the radial projection angle, similar to a stack‐of‐stars acquisition [56]. Advancements in motion correction techniques that rely on developments in RF coil hardware [57], machine learning models [58], or external tracking systems [59] may also be adapted to mitigate motion sensitivity.

The minimum TE of the δSB‐UTE and δMB‐UTE sequences is limited by the duration of the CAIPI gradient. The area of the gradient scales inversely with slice separation, resulting in a longer CAIPI gradient duration for smaller slice separations. In this work, the gradient was 0.089 and 0.125 ms for 40‐ and 20‐mm slice separations, respectively. These durations were shorter than the time required for the RF coils to switch from transmit to receive and did not limit the minimum TE.

For structures that contain multiple spin populations, longer *T*_2_ signals will be more heavily weighted in images obtained with the δSB‐UTE and δMB‐UTE sequences (Figures 2 and S3). For example, bone contains spin populations with *T*_2_ = 4 ms [5, 6, 7, 8] that are expected to be excited nearly twice as effectively as spin populations with T_2_ = 0.3 ms [5, 6, 7, 8]. For imaging even shorter T_2_ signals, such as that of non‐aqueous myelin (*T*_2_ = 5.5 and 100 μs [60]), the δSB‐UTE and δMB‐UTE sequences are expected to be ineffective as very little signal will be excited. However, the sequences may be well‐suited for imaging tendons (*T*_2_ = 1 and 20 ms [1, 2, 3, 4]) and ligaments (*T*_2_ = 1 and 14 ms [3, 4]). For the δMB‐UTE sequence, the weighting of short‐ and long‐ T_2_ signals differs between the slices due to short‐T_2_‐related broadening. Although the outer slices experience increased broadening, the inner slices have a reduced amount of short‐T_2_ signal due to the overlap of broadened saturation blocks. This effect may also impact longer *T*_2_ signals via magnetization transfer and is illustrated by the mean values in Table S5.

### 4.3 Design Considerations

To reduce the impact of short‐T_2_ slice profile broadening, prepulses may be designed with shorter durations (while maintaining the same TBW). The duration of the prepulse can be reduced by decreasing *α*_pre_, which results in reduced power deposition (enabling the use of shorter TRs) and reduced signal within the slices. In this work, *α*_pre_ = 70° was used to balance the trade‐offs between SNR and scan time; however, *α*_pre_ may be decreased to capture shorter *T*_2_ signals or increased to increase SNR. Prepulse duration can also be decreased by reducing prepulse TBW, which results in a decrease in power deposition and broadening of slice profiles. In this work, relatively sharp slices were obtained with prepulse TBWs of 13.2 and 6.6 for the δSB‐UTE and δMB‐UTE sequences, respectively.

Although the use of shorter TRs results in shorter scan times, signal within the slices is decreased and slices shift away from their desired locations. In this work, Ernst angle excitation was used to increase the amount of signal surrounding the saturation blocks and improve SNR. The shifting of slices at shorter TRs may result in aliasing artifacts. Slice position error may be counteracted by designing prepulses with a smaller saturation block thickness and a larger separation.

The through‐plane FOV is given by 2 and 4 times the saturation block thickness for the δSB‐UTE and δMB‐UTE sequences, respectively. To image a larger FOV, saturation block thickness can be increased at the cost of broader slices. The use of a saturation block thickness of 40 and 20 mm for the δSB‐UTE and δMB‐UTE sequences, respectively, enabled the imaging of an 80 mm through‐plane FOV with relatively sharp slices. The through‐plane resolution is determined by the saturation block shift, with larger shifts resulting in thicker slices.

### 4.4 Future Applications

The δSB‐UTE and δMB‐UTE sequences may reduce scan times for application to transcranial focused ultrasound [61], in which only a portion of the skull may need to be mapped for treatment planning. In addition, the sequences may be modified to image short‐T_2_ signals from sodium (^23^Na) in the extremities, where multi‐slice imaging would enhance studies of cardiovascular disease mechanisms [62].

## 5 Conclusion

The δSB‐UTE and δMB‐UTE sequences were designed and implemented in vivo at 3 T to increase flexibility in FOV and reduce scan times for imaging short‐T_2_ spin populations. Slice profiles were investigated with various prepulse flip angles, TRs, prepulse TBWs, saturation block thicknesses, and saturation block shifts. The δSB‐UTE and δMB‐UTE sequences were used to image a 210 × 210 × 80 mm^3^ FOV with 1.0 × 1.0 × 4.0 mm resolution in 2.66 and 1.45 min, respectively. Even if the same FOV was of interest, a 3D UTE sequence with a kooshball acquisition and TR of 5 ms would require 6.77 min to image a 210 mm isotropic FOV with the same voxel volume. With the acquisition parameters used in this work, the required scan time of the δSB‐UTE and δMB‐UTE sequences is 3.64 and 6.67 times less than half‐pulse UTE sequences, respectively. The δSB‐UTE and δMB‐UTE sequences may enable the rapid imaging of short‐T_2_ signals from tendons, ligaments, and bone with a reduced FOV and a TE of 0.18 ms.

## Funding

This work was supported by the Natural Sciences and Engineering Council of Canada (NSERC), Discovery Grant, RGPIN‐2020‐06005; Natural Sciences and Engineering Council of Canada (NSERC), Canada Graduate Scholarships—Doctoral program; Natural Sciences and Engineering Council of Canada (NSERC), Canada Graduate Scholarships—Michael Smith Foreign Study Supplements; Michael Smith Health Research BC (MSHRBC) Scholar Program, SCH‐2023‐3224; Canadian Foundation for Innovation (CFI), John R. Evans Leaders Fund, 40398; University of British Columbia (UBC), GoGlobal Self Directed Research Abroad Award; National Institutes of Health (NIH), Grants, 1S10OD021771‐01, R01HL155523, and R01HL157378; National Center for Advancing Translational Sciences (NCATS), Clinical Translational Science Award (CTSA) Program, UL1 TR000445.

## Supporting information


**Table S1:** Parameters of the δSB‐UTE and δMB‐UTE sequences used for phantom slice profile experiments. The parameters changed for each experiment are italicized.
**Table S2:** Parameters of the δSB‐UTE and δMB‐UTE sequences used for phantom imaging.
**Table S3:** Parameters of the δSB‐UTE and δMB‐UTE sequences used for in vivo experiments.
**Table S4:** Parameters of the δSB‐UTE and δMB‐UTE sequences used for in vivo motion tolerance experiments.
**Table S5:** Mean signal, SNR, CNR, and edge sharpness for images obtained with the δSB‐UTE, δMB‐UTE, and 3D UTE sequences under matched voxel volumes and scan times (in vivo experiment 3).
**Table S6:** Artifact power for the motion tolerance experiments performed with the δSB‐UTE sequence.
**Table S7:** Artifact power for the motion tolerance experiments performed with the δMB‐UTE sequence.
**Figure S1:** A schematic diagram of the δSB‐UTE sequence showing transverse magnetization profiles throughout the sequence and the subtraction of subsequent acquisitions. In the first acquisition a single‐block prepulse (blue) tips a wide region of magnetization into the transverse plane (blue) and spoiler gradients dephase the transverse magnetization. A non‐selective rectangular pulse (red) excites the surrounding magnetization (purple) and data acquisition begins following the CAIPI gradient and transmit/receive switching. In the second acquisition, frequency modulation of the prepulse (yellow) shifts the saturation blocks (yellow, orange) along the slice‐select direction. Subtraction of subsequent acquisitions enables the reconstruction of two (I and II) simultaneous slices.
**Figure S2:** The RF waveform (blue), prescribed gradient (red, dashed), and expected gradient (red, solid) of 70° single‐block (A) and multi‐block (B) prepulses. The single‐block prepulse has a TBW of 13.2 and saturation block thickness of 40 mm, while the multi‐block prepulse has a TBW of 6.6 and saturation block thickness of 20 mm.
**Figure S3:** Slice profiles of δSB‐UTE sequence simulated with T_2_ = 0.1–10 ms. Parameters included a prepulse flip angle of 70°, prepulse TBW of 13.2, saturation block thickness of 40 mm, TR of 35 ms, excitation flip angle of 29.9°, and *T*_1_ of 245 ms. The saturation block was not shifted to produce saturation block profiles (A) and was shifted of 4 mm to produce slice profiles (B). The total signal and profile maximum (C) and FWHM (D) are plotted for each slice.
**Figure S4:** Slice profiles of δSB‐UTE sequence simulated with TR = 30–150 ms. Parameters included a prepulse flip angle of 70°, prepulse TBW of 13.2, saturation block thickness of 40 mm, excitation flip angles of 27.8°–57.2°, *T*_2_ of 5 ms, and *T*_1_ of 245 ms. The saturation block was not shifted to produce saturation block profiles (A) and was shifted of 4 mm to produce slice profiles (B). The outward shift of the slices away from their expected positions of ±20 mm is plotted (C).
**Figure S5:** Slice profiles of δMB‐UTE sequence simulated with TR = 30–150 ms. Parameters included a prepulse flip angle of 70°, prepulse TBW of 6.6, saturation block thickness of 20 mm, excitation flip angles of 27.8°–57.2°, *T*_2_ of 5 ms, and *T*_1_ of 245 ms. The saturation block was not shifted to produce saturation block profiles (A) and was shifted of 4 mm to produce slice profiles (B). The outward shift of the slices away from their expected positions of ±10 mm and ±30 mm is plotted (C).
**Figure S6:** Simulated longitudinal magnetization profiles after a single iteration of a single‐block prepulse for off‐resonance frequencies of −800 to 800 Hz (A) and B_1_ scale factors of 0.4–1.6 (B). Parameters included a prepulse flip angle of 70°, TBW of 13.2, saturation block thickness of 40 mm, *T*_2_ of 5 ms, and *T*_1_ of 245 ms.
**Figure S7:** Simulated longitudinal magnetization profiles after a single iteration of a multi‐block prepulse for off‐resonance frequencies of −800 to 800 Hz (A) and B_1_ scale factors of 0.4–1.6 (B). Parameters included a prepulse flip angle of 70°, TBW of 6.6, saturation block thickness of 20 mm, *T*_2_ of 5 ms, and *T*_1_ of 245 ms.
**Figure S8:** Results of varying prepulse flip angle and TR (phantom experiment 1) for the δSB‐UTE sequence. Saturation block and slice projections obtained with prepulse flip angles of 40, 70°, and 110° and a TR of 150 ms and a prepulse flip angle of 70° (A). Slice profiles obtained with TRs of 33, 53, and 150 ms (B). Plots of total signal (solid) and profile maximum (dashed) (C) and FWHM (D) for prepulse flip angles of 30°–120° and TRs of 53 and 150 ms. Other parameters included a saturation block thickness of 40 mm, prepulse TBW of 13.2, saturation block shift of 4 mm, and excitation flip angles of 25.7°–51.5°.
**Figure S9:** Results of varying prepulse TBW (phantom slice profile experiment 2) for the δSB‐UTE sequence. Saturation block and slice projections (A) and corresponding slice profiles (B) obtained with prepulse TBWs 6.6, 13.2, and 19.8. Total signal, profile maximum, and FWHM are tabulated for each TBW. Other parameters included a prepulse flip angle of 70°, TR of 53 ms, saturation block thickness of 40 mm, saturation block shift of 4 mm, and excitation flip angle of 32.2°.
**Figure S10:** Results of varying prepulse TBW (phantom slice profile experiment 2) for the δMB‐UTE sequence. Saturation block and slice projections (A) and corresponding slice profiles (B) obtained with prepulse TBWs 3.3, 6.6, and 9.9. Total signal, profile maximum, and FWHM are tabulated for each TBW. Other parameters included a prepulse flip angle of 70°, TR of 53 ms, saturation block thickness of 20 mm, saturation block shift of 4 mm, and excitation flip angle of 32.2°.
**Figure S11:** Results of varying saturation block thickness (phantom slice profile experiment 3) for the δSB‐UTE sequence. Saturation block and slice projections (A) and corresponding slice profiles (B) obtained with saturation block thicknesses of 20, 40, and 60 mm. Total signal, profile maximum, and FWHM are tabulated for each thickness. Other parameters included a prepulse flip angle of 70°, prepulse TBW of 13.2, TR of 53 ms, saturation block shift of 4 mm, and excitation flip angle of 32.2°.
**Figure S12:** Results of varying saturation block thickness (phantom slice profile experiment 3) for the δMB‐UTE sequence. Saturation block and slice projections (A) and corresponding slice profiles (B) obtained with saturation block thicknesses of 10, 20, and 40 mm. Total signal, profile maximum, and FWHM are tabulated for each thickness. Other parameters included a prepulse flip angle of 70°, prepulse TBW of 6.6, TR of 53 ms, saturation block shift of 4 mm, and excitation flip angle of 32.2°.
**Figure S13:** Results of varying saturation block shift (phantom slice profile experiment 4) for the δSB‐UTE sequence. Slice projections (A) and corresponding slice profiles (B) obtained with saturation block shifts of 1–10 mm. Plots of total signal (solid) and profile maximum (dashed) (C) and FWHM (D) for each shift. Other parameters included a prepulse flip angle of 70°, prepulse TBW of 13.2, TR of 53 ms, saturation block thickness of 40 mm, and excitation flip angle of 32.2°.
**Figure S14:** Results of varying saturation block shift (phantom slice profile experiment 4) for the δMB‐UTE sequence. Slice projections (A) and corresponding slice profiles (B) obtained with saturation block shifts of 1–10 mm. Plots of total signal (solid) and profile maximum (dashed) (C) and FWHM (D) for each shift. Other parameters included a prepulse flip angle of 70°, prepulse TBW of 6.6, TR of 53 ms, saturation block thickness of 20 mm, and excitation flip angle of 32.2°.
**Figure S15:** Sagittal images (A) and slice projections (B) obtained with the δSB‐UTE sequence over an 80 mm through‐plane FOV in a phantom. The images in sets I and II correspond to the simultaneous slices below. Parameters included a prepulse flip angle of 70°, prepulse TBW of 13.2, saturation block thickness of 40 mm, saturation block shift of 4 mm, TR of 31 ms, excitation flip angle of 24.9°, TE of 0.17 ms, and 11 acquisitions. The scan time for imaging was 2.42 min.
**Figure S16:** Sagittal images (A) and slice projections (B) obtained with the δMB‐UTE sequence over an 80 mm through‐plane FOV in a phantom. The images in sets I, II, III, and IV correspond to the simultaneous slices below. Parameters included a prepulse flip angle of 70°, prepulse TBW of 6.6, saturation block thickness of 20 mm, saturation block shift of 4 mm, TR of 31 ms, excitation flip angle of 24.9°, TE of 0.17 ms, and 6 acquisitions. The scan time for imaging was 1.33 min.
**Figure S17:** In vivo slice projections (A) and profiles (B) obtained using the δSB‐UTE sequence with TE_1_ = 0.18 ms and TE_2_ = 2.34 ms (in vivo experiment 1). Slices I and II correspond to the axial images in Figure 5A. Parameters included a prepulse flip angle of 70°, prepulse TBW of 13.2, saturation block thickness of 40 mm, saturation block shift of 4 mm, and TR of 34 ms, excitation flip angle of 29.5°.
**Figure S18:** ROIs used to calculate SNR, CNR, and edge sharpness for images obtained with the δSB‐UTE, δMB‐UTE, and 3D UTE sequences under matched voxel volumes and scan times (in vivo experiment 3). The bone (blue), brain tissue (yellow), and edge (red) ROIs are overlaid on zoomed regions of the images obtained with the 3D UTE sequence.
**Figure S19:** Results of sagittal (A) and axial (B) in vivo motion tolerance tests performed with the δSB‐UTE sequence. Images obtained without motion, with a few millimeter‐pitch nodding (motion 1), a few centimeter‐pitch shift in the foot‐head direction (motion 2), and with mouth and eye movement (motion 3). Parameters included a prepulse flip angle of 70°, prepulse TBW of 13.2, saturation block thickness of 40 mm, saturation block shift of 4 mm, and TR of 34 ms, excitation flip angle of 29.5°.
**Figure S20:** Results of sagittal in vivo motion tolerance tests performed with the δMB‐UTE sequence. Images obtained without motion, with a few millimeter‐pitch nodding (motion 1), a few centimeter‐pitch shift in the foot‐head direction (motion 2), and with mouth and eye movement (motion 3). Parameters included a prepulse flip angle of 70°, prepulse TBW of 13.2, saturation block thickness of 40 mm, saturation block shift of 4 mm, and TR of 34 ms, excitation flip angle of 29.5°.
**Figure S21:** Results of axial in vivo motion tolerance tests performed with the δMB‐UTE sequence. Images obtained without motion, with a few millimeter‐pitch nodding (motion 1), a few centimeter‐pitch shift in the foot‐head direction (motion 2), and with mouth and eye movement (motion 3). Parameters included a prepulse flip angle of 70°, prepulse TBW of 13.2, saturation block thickness of 40 mm, saturation block shift of 4 mm, and TR of 34 ms, excitation flip angle of 29.5°.
**Figure S22:** L‐factor and g‐factor maps for echo 1 (A and B, respectively) and echo 2 (C and D, respectively) of the δSB‐UTE sequence. The acceleration factor of the acquisition used for the δSB‐UTE sequence was 3.1 relative to the fully‐sampled reference acquisition.
**Figure S23:** L‐factor and g‐factor maps for echo 1 (A and B, respectively) and echo 2 (C and D, respectively) of the δMB‐UTE sequence. The acceleration factor of the acquisition used for the δMB‐UTE sequence was 6.3 relative to the fully‐sampled reference acquisition.

## Data Availability

The data that support the findings of this study are available from Philips. Restrictions apply to the availability of these data, which were used under license for this study. Data are available from the author(s) with the permission of Philips.
