# Supplementary material for: Design of Differential Single‐Block and Multi‐Block Presaturated Ultrashort Echo Time Pulse Sequences for Fast and Flexible Short‐T2 Imaging
Source: Magn Reson Med. 2026 Apr 20;96(2):711–26. doi: 10.1002/mrm.70379 (PMC13269236; doi:10.1002/mrm.70379)
Supplement: Supplementary file 1 — Table S1: Parameters of the δSB‐UTE and δMB‐UTE sequences used for phantom slice profile experiments. The parameters changed for each experiment are italicized. Table S2: Parameters of the δSB‐UTE and δMB‐UTE sequences used for phantom imaging. Table S3: Parameters of the δSB‐UTE and δMB‐UTE sequences used for in vivo experiments. Table S4: Parameters of the δSB‐UTE and δMB‐UTE sequences used for in vivo motion tolerance experiments. Table S5: Mean signal, SNR, CNR, and edge sharpness for images obtained with the δSB‐UTE, δMB‐UTE, and 3D UTE sequences under matched voxel volumes and scan times (in vivo experiment 3). Table S6: Artifact power for the motion tolerance experiments performed with the δSB‐UTE sequence. Table S7: Artifact power for the motion tolerance experiments performed with the δMB‐UTE sequence. Figure S1: A schematic diagram of the δSB‐UTE sequence showing transverse magnetization profiles throughout the sequence and the subtraction of subsequent acquisitions. In the first acquisition a single‐block prepulse (blue) tips a wide region of magnetization into the transverse plane (blue) and spoiler gradients dephase the transverse magnetization. A non‐selective rectangular pulse (red) excites the surrounding magnetization (purple) and data acquisition begins following the CAIPI gradient and transmit/receive switching. In the second acquisition, frequency modulation of the prepulse (yellow) shifts the saturation blocks (yellow, orange) along the slice‐select direction. Subtraction of subsequent acquisitions enables the reconstruction of two (I and II) simultaneous slices. Figure S2: The RF waveform (blue), prescribed gradient (red, dashed), and expected gradient (red, solid) of 70° single‐block (A) and multi‐block (B) prepulses. The single‐block prepulse has a TBW of 13.2 and saturation block thickness of 40 mm, while the multi‐block prepulse has a TBW of 6.6 and saturation block thickness of 20 mm. Figure S3: Slice profiles of δSB‐UTE sequence simulated [file MRM-96-711-s001.docx]

Design of differential single-block and multi-block presaturated ultrashort echo time pulse sequences for fast and flexible short-T­_2_ imaging

Jason A. Reich^1^, Shannon L. Taylor^2,3^, Kevin D. Harkins^2,3,4^, Rachelle L. Crescenzi^2,3,4^, Erin L. MacMillan^5,6^, Rebecca E. Feldman^1,7^

^1^Department of Computer Science, Mathematics, Physics and Statistics, University of British Columbia, Kelowna, British Columbia, Canada

^2^Biomedical Engineering, Vanderbilt University, Nashville, TN, United States

^3^Vanderbilt University Institute of Imaging Science, Vanderbilt University Medical Center, Nashville, TN, United States

^4^Radiology and Radiological Sciences, Vanderbilt University Medical Center, Nashville, TN, United States

^5^UBC MRI Research Centre, Department of Radiology, University of British Columbia, Vancouver, British Columbia, Canada

^6^Djavad Mowafaghian Centre for Brain Health, University of British Columbia, Vancouver, British Columbia, Canada

^7^Biomedical Engineering and Imaging Institute, Icahn School of Medicine at Mount Sinai, New York, New York, United States of America

**Supplemental Material**

**Supporting Information:**

**Information S1. Pulse Sequence Design**

The δSB and δMB-UTE sequences were designed to meet hardware specifications including a dwell time of 6.4 or 8.0 µs, maximum B­_1_ of 22 µT, maximum gradient strength of 22.5 mT/m/ms, and maximum slew rate of 150 mT/m/ms. Prepulses designed with the Multiband RF Toolbox used least squares optimization, linear phase, and in-slice and out-of-slice ripples of 0.01 that were calculated for a saturation profile^1^. Prepulses were shortened with a single iteration of the VERSE algorithm using a gradient impulse response function provided in the Multiband RF Toolbox. The duration of the spoiler gradients, whole-volume excitation, and CAIPI gradient were minimized by using the maximum B_1_­, gradient strength, and slew rate.

**Information S2. SAR Accounting**

Relative SAR was calculated for each prepulse according to

$$SAR\propto\sum_{i=1}^{N} \left| B_{1,i} \right|^{2}\Delta t,$$

where $\Delta t$ is the dwell time, $N$ is the number of samples in the RF waveform, and $B_{1,i}$ is the amplitude of the $i^{th}$ sample of the RF waveform.

**Information S3. Phantom Imaging**

Sagittal images and projections obtained from the PIQT phantom were acquired over an 80 mm through-plane FOV. Both sequences used α_pre_ = 70º, TR = 31 ms, α_ex_ = 24.9º, TE = 0.17 ms, and spoiler gradients on all axes. The δSB-UTE sequence used a prepulse TBW of 13.2, a saturation block thickness of 40 mm, and 11 acquisitions with saturation block shifts of 4 mm to acquire 20 slices over 80 mm. The δMB-UTE sequence used a prepulse TBW of 6.6, a saturation block thickness of 20 mm, and 6 acquisitions with saturation block shifts of 4 mm to acquire 20 slices over 80 mm. The CAIPI gradient was used for the acquisition of images but not slice projections. All other parameters were the same as described for slice profile investigation (see Table S2). The scan times required by the δSB-UTE and δMB-UTE sequences were 2.42 and 1.33 min, respectively. Slice projections were reconstructed as described for phantom experiments 1-4 and images were reconstructed as described for *in-vivo* experiments 1 and 2.

**Information S4. Motion Tolerance Experiments**

Motion tolerance tests were performed in axial and sagittal orientations with the same parameters as for the acquisition of axial *in-vivo* images, except: N_ro_ = 480, N_s_ = 265/480 (TE_1_/TE_2_), and FOV = 240x240 mm^2^ (see Table S4). In addition to a motion-free reference, three motion regimes were investigated: (1) The participant was instructed to nod their head with a few mm pitch a few times during the acquisition. (2) The participant was instructed to shift a few cm in the foot-head direction, then return to their original position, once during the acquisition. (3) The participant was instructed to yawn twice and move their eyes throughout the acquisition. Images from motion tolerance tests were reconstructed with an in-plane resolution of 0.71x0.71 mm^2^ as described for *in-vivo* experiments 1 and 2. For each motion regime, the resulting images ($I^{motion})$were rigidly coregisterred to the motion-free reference image ($I^{reference})$and artifact power was quantified according to^2^

$$Artifact Power= \frac{\sum_{j=1}^{N} \left| \left| I_{j}^{reference} \right|-\left| I_{j}^{motion} \right| \right|^{2}}{\sum_{j=1}^{N} \left| I_{j}^{reference} \right|^{2}},$$

where $j$ is the voxel index and $N$ is the number of voxels (see Table S6 and S7).

**Information S5. Slice Leakage and Noise Amplification**

Slice leakage and noise amplification maps were calculated using the motion-free reference from the motion tolerance experiment. Slice leakage was quantified with L-factor maps^3^ calculated with data that was synthesized as described previously^4^. For each modulation frequency, images were normalized by the sum of the magnitude image with the largest signal (the slice that was modulated at a given frequency befor reconstruction). Noise amplification was quantified with g-factor maps calculated using Monte-Carlo simulations^5^ with 10000 instances. The undersampled image was reconstructed as described for the motion tolerance experiment. For the noise-only reference, N_ro_ = 754, resulting in an acceleration factor of 3.1 and 6.3 for the δSB-UTE and δMB-UTE sequences respectively. Noise-only images were reconstructed to the same resolution as undersampled images with the “*fft*” and “*nufft*” commands in BART.

**References:**

1. Pauly J, Nishimura D, Macovski A, Roux P Le. Parameter relations for the Shinnar-Le Roux selective excitation pulse design algorithm. *IEEE Trans Med Imaging*. 1991;10(1):53-65. doi:10.1109/42.75611

2. Xiao Z, Hoge WS, Mulkern R V., Zhao L, Hu G, Kyriakos WE. Comparison of parallel MRI reconstruction methods for accelerated 3D fast spin-echo imaging. *Magn Reson Med*. 2008;60(3):650-660. doi:10.1002/mrm.21679

3. Xu J, Moeller S, Auerbach EJ, et al. Evaluation of slice accelerations using multiband echo planar imaging at 3T. *Neuroimage*. 2013;83:991-1001. doi:10.1016/j.neuroimage.2013.07.055

4. Risk BB, Kociuba MC, Rowe DB. Impacts of simultaneous multislice acquisition on sensitivity and specificity in fMRI. *Neuroimage*. 2018;172:538-553. doi:10.1016/j.neuroimage.2018.01.078

5. Robson PM, Grant AK, Madhuranthakam AJ, Lattanzi R, Sodickson DK, McKenzie CA. Comprehensive quantification of signal-to-noise ratio and g-factor for image-based and k-space-based parallel imaging reconstructions. *Magn Reson Med*. 2008;60(4):895-907. doi:10.1002/mrm.21728

**Supporting Tables:**

**Table S1:** Parameters of the δSB-UTE and δMB-UTE sequences used for phantom slice profile experiments. The parameters changed for each experiment are italicized.

| **Experiment # / Parameter Changed** | **1 / Prepulse Flip Angle and TR** | | **2 / Prepulse TBW** | | **3 / Saturation Block Thickness** | | **4 / Saturation Block Shift** | |
| --- | --- | --- | --- | --- | --- | --- | --- | --- |
| **Sequence** | δSB | δMB | δSB | δMB | δSB | δMB | δSB | δMB |
| **Prepulse Flip Angle, α_pre_ [º]** | *30, 40, 50, 60, 70, 80, 90, 100, 110, 120* | | 70 | | 70 | | 70 | |
| **Prepulse TBW** | 13.2 | 6.6 | *6.6, 13.2, 19.8* | *3.3, 6.6, 9.9* | 13.2 | 6.6 | 13.2 | 6.6 |
| **Saturation Block Thickness [mm]** | 40 | 20 | 40 | 20 | *20, 40, 60* | *10, 20, 30* | 40 | 20 |
| **Prepulse Duration [µs]** | *742, 794, 838, 883, 928, 986, 1050, 1114, 1190, 1306* | *774, 845,*  *915, 998,*  *1094,1190,*  *1306, 1414,*  *1536, 1651* | *698, 928, 1110* | *800, 1094, 1299* | *1165, 928,*  *870* | *1312, 1094, 1082* | 928 | 1094 |
| **Dwell Time [µs]** | 6.4 | 6.4 | 6.4 | 6.4 | 6.4 | 6.4 | 6.4 | 6.4 |
| **Relative Prepulse SAR [µT^2^·ms]** | *46.9, 65.0, 85.7, 108.2, 131.2, 152.5, 173.6, 195.2, 216.5, 229.4* | *48.8, 69.7, 91.4, 111.0, 130.7, 150.4, 167.7, 186.1, 205.2, 225.4* | *112.3, 131.2, 141.5* | *109.5, 130.7, 140.4* | *127.8, 131.2, 123.5* | *124.6, 130.7, 121.9* | 131.2 | 130.7 |
| **Saturation Block Shift [mm]** | 4 | | 4 | | 4 | | *1, 2, 3, 4, 5, 6, 7, 8, 9, 10* | |
| **TR [ms]** | *33, 53, 150* | | 53 | | 53 | | 53 | |
| **Excitation Flip Angle, α_ex_ [º]** | *25.7, 32.2, 51.5* | | 32.2 | | 32.2 | | 32.2 | |
| **Excitation Duration [µs]** | *77, 96, 154* | | 96 | | 96 | | 96 | |
| **TE [ms]** | *0.18, 0.18, 0.21* | | 0.18 | | 0.18 | | 0.18 | |
| **Radial Projections** | 424 | | 424 | | 424 | | 424 | |
| **Readout (Ramp) Samples** | 253 (83) | | 253 (83) | | 253 (83) | | 253 (83) | |
| **In-plane FOV [mm]** | 210x210 | | 210x210 | | 210x210 | | 210x210 | |
| **Acquired In-plane Resolution [mm]** | 1.00x1.00 | | 1.00x1.00 | | 1.00x1.00 | | 1.00x1.00 | |
| **Reconstructed In-plane Resolution [mm]** | 0.73x0.73 | | 0.73x0.73 | | 0.73x0.73 | | 0.73x0.73 | |

**Table S2:** Parameters of the δSB-UTE and δMB-UTE sequences used for phantom imaging.

| **Experiment** | **Images** | | **Slice Projections** | |
| --- | --- | --- | --- | --- |
| **Sequence** | δSB | δMB | δSB | δMB |
| **Prepulse Flip Angle, α_pre_ [º]** | 70 | | 70 | |
| **Prepulse TBW** | 13.2 | 6.6 | 13.2 | 6.6 |
| **Saturation Block Thickness [mm]** | 40 | 20 | 40 | 20 |
| **Prepulse Duration [µs]** | 928 | 1094 | 928 | 1094 |
| **Dwell Time [µs]** | 6.4 | 6.4 | 6.4 | 6.4 |
| **Relative Prepulse SAR [µT^2^·ms]** | 131.2 | 130.7 | 131.2 | 130.7 |
| **Saturation Block Shift [mm]** | 4 | | 4 | |
| **TR [ms]** | 31 | | 31 | |
| **Excitation Flip Angle, α_ex_ [º]** | 24.9 | | 24.9 | |
| **Excitation Duration [µs]** | 75 | | 75 | |
| **TE [ms]** | 0.17 | | 0.17 | |
| **Radial Projections** | 424 | | 424 | |
| **Echo 1, 2 Readout (Ramp) Samples** | 253 (83),  - | | 253 (83),  - | |
| **FOV [mm]** | 210x210 | | 210x210 | |
| **Acquired In-plane Resolution [mm]** | 1.00x1.00 | | 1.00x1.00 | |
| **Reconstructed In-plane Resolution [mm]** | 0.73x0.73 | | 0.73x0.73 | |
| **Scan Time [min]** | 2.42 | 1.33 | 2.42 | 1.33 |

**Table S3:** Parameters of the δSB-UTE and δMB-UTE sequences used for *in-vivo* experiments.

| **Experiment # / Description** | **1 / Axial *In-vivo* Images** | | **1 / Axial *In-vivo* Slice Projections** | | **2 / Sagittal *In-vivo* Images** | | **3 / 3D UTE Comparison Images** | | |
| --- | --- | --- | --- | --- | --- | --- | --- | --- | --- |
| **Sequence** | δSB | δMB | δSB | δMB | δSB | δMB | δSB | δMB | 3D |
| **Prepulse Flip Angle, α_pre_ [º]** | 70 | | 70 | | 70 | | 70 | | - |
| **Prepulse TBW** | 13.2 | 6.6 | 13.2 | 6.6 | 13.2 | 6.6 | 13.2 | 6.6 | - |
| **Saturation Block Thickness [mm]** | 40 | 20 | 40 | 20 | 40 | 20 | 40 | 20 | - |
| **Prepulse Duration [µs]** | 928 | 1088 | 928 | 1088 | 928 | 1088 | 928 | 1088 | - |
| **Dwell Time [µs]** | 6.4 | 6.4 | 6.4 | 6.4 | 6.4 | 6.4 | 8.0 | 8.0 | 8.0 |
| **Relative Prepulse SAR [µT^2^·ms]** | 131.2 | 130.7 | 131.2 | 130.7 | 131.2 | 130.7 | 130.9 | 130.3 | - |
| **Saturation Block Shift [mm]** | 4 | | 4 | | 4 | | 4 | | - |
| **TR [ms]** | 34 | | 34 | | 34 | | 34 | | |
| **Excitation Flip Angle, α_ex_ [º]** | 29.5 | | 29.5 | | 29.5 | | 29.5 | | |
| **Excitation Duration [µs]** | 88 | | 88 | | 88 | | 88 | | |
| **Echo 1, 2 TE [ms]** | 0.18, 2.34 | | 0.18, 2.34 | | 0.18, 2.34 | | 0.18, 2.34 | | |
| **Radial Projections** | 400 | | 360 | | 424 | | 448 | | 29,540 |
| **Echo 1, 2 Readout (Ramp) Samples** | 253 (107), 400 (0) | | 216 (73),  360 (0) | | 234 (44),  424 (0) | | 247 (47),  448 (0) | | 163 (47), 280 (0) |
| **Number of Scan Averages** | 1 | | 1 | | 1 | | 6 | 11 | 1 |
| **FOV [mm]** | 200x200 | | 180x180 | | 210x210 | | 220x220 | | 220x  220x  220 |
| **Acquired Resolution [mm]** | 1.00x1.00 | | 1.00x1.00 | | 1.00x1.00 | | 0.98x0.98 | | 1.57x  1.57x  1.57 |
| **Reconstructed Resolution [mm]** | 0.69x0.69 | | 0.70x0.70 | | 0.73x0.73 | | 0.69x0.69 | | 0.69x  0.69x  4.00 |
| **Scan Time [min]** | 0.42 | 0.42 | 0.47 | 0.47 | 2.66 | 1.45 | 3.07 | 5.60 | 16.75 |

**Table S4:** Parameters of the δSB-UTE and δMB-UTE sequences used for *in-vivo* motion tolerance experiments.

| **Experiment # / Description** | **1 / Nodding with Few Millimeter Pitch** | | | **2 / Few Centimeter Shift in FH** | | **3 / Mouth and Eye Movement** | |
| --- | --- | --- | --- | --- | --- | --- | --- |
| **Sequence** | δSB | δMB | | δSB | δMB | δSB | δMB |
| **Prepulse Flip Angle, α_pre_ [º]** | 70 | | | 70 | | 70 | |
| **Prepulse TBW** | 13.2 | | 6.6 | 13.2 | 6.6 | 13.2 | 6.6 |
| **Saturation Block Thickness [mm]** | 40 | | 20 | 40 | 20 | 40 | 20 |
| **Prepulse Duration [µs]** | 928 | | 1088 | 928 | 1088 | 928 | 1088 |
| **Dwell Time [µs]** | 8.0 | 8.0 | | 8.0 | 8.0 | 8.0 | 8.0 |
| **Relative Prepulse SAR [µT·ms]** | 130.9 | 130.3 | | 130.9 | 130.3 | 130.9 | 130.3 |
| **Saturation Block Shift [mm]** | 4 | | | 4 | | 4 | |
| **TR [ms]** | 34 | | | 34 | | 34 | |
| **Excitation Flip Angle, α_ex_ [º]** | 29.5 | | | 29.5 | | 29.5 | |
| **Excitation Duration [µs]** | 88 | | | 88 | | 88 | |
| **Echo 1, 2 TE [ms]** | 0.18, 2.34 | | | 0.18, 2.34 | | 0.18, 2.34 | |
| **Radial Projections** | 480 | | | 480 | | 480 | |
| **Echo 1, 2 Readout (Ramp) Samples** | 265 (51), 480 (0) | | | 265 (51), 480 (0) | | 265 (51), 480 (0) | |
| **FOV [mm]** | 240x240 | | | 240x240 | | 240x240 | |
| **Acquired In-plane Resolution [mm]** | 1.00x1.00 | | | 1.00x1.00 | | 1.00x1.00 | |
| **Reconstructed In-plane Resolution [mm]** | 0.71x0.71 | | | 0.71x0.71 | | 0.71x0.71 | |

**Table S5:** Mean signal, SNR, CNR, and edge sharpness for images obtained with the δSB-UTE, δMB-UTE, and 3D UTE sequences under matched voxel volumes and scan times (*in-vivo* experiment 3).

| **Echo 1** | | | | | | | | | | |
| --- | --- | --- | --- | --- | --- | --- | --- | --- | --- | --- |
| **Sequence** | **3D UTE** | | | | **δSB-UTE** | | **δMB-UTE** | | | |
| **Slice** | **I** | **II** | **III** | **IV** | **II** | **IV** | **I** | **II** | **III** | **IV** |
| **Mean** | 0.96 | 0.89 | 0.87 | 1.00 | 0.74 | 1.00 | 0.79 | 0.66 | 0.66 | 1.00 |
| **SNR** | 347.1 | 287.4 | 201.1 | 299.5 | 65.7 | 66.4 | 60.6 | 64.9 | 71.6 | 69.3 |
| **CNR** | 141.3 | 119.4 | 121.9 | 103.3 | 22.4 | 21.0 | 27.4 | 29.7 | 40.2 | 25.7 |
| **Edge Sharpness** | 0.108 | 0.111 | 0.083 | 0.126 | 0.148 | 0.066 | 0.081 | 0.162 | 0.086 | 0.082 |
| **Echo 2** | | | | | | | | | | |
| **Sequence** | **3D UTE** | | | | **δSB-UTE** | | **δMB-UTE** | | | |
| **Slice** | **I** | **II** | **III** | **IV** | **II** | **IV** | **I** | **II** | **III** | **IV** |
| **Mean** | 0.76 | 0.70 | 0.69 | 0.80 | 0.58 | 0.71 | 0.61 | 0.53 | 0.51 | 0.69 |
| **SNR** | 252.4 | 226.3 | 247.5 | 293.5 | 53.3 | 40.0 | 55.1 | 45.8 | 47.8 | 58.4 |
| **CNR** | 109.8 | 43.4 | 68.8 | 60.9 | 11.8 | 17.0 | 16.4 | 19.1 | 17.9 | 17.8 |
| **Edge Sharpness** | 0.114 | 0.104 | 0.071 | 0.123 | 0.131 | 0.100 | 0.085 | 0.136 | 0.067 | 0.098 |

**Table S6:** Artifact power for the motion tolerance experiments performed with the δSB-UTE sequence.

| **Sagittal** | | | | |
| --- | --- | --- | --- | --- |
| **Echo** | **1** | | **2** | |
| **Slice** | **I** | **II** | **I** | **II** |
| **Motion 1** | 0.120 | 0.166 | 0.113 | 0.149 |
| **Motion 2** | 1.270 | 0.957 | 3.055 | 4.217 |
| **Motion 3** | 0.102 | 0.102 | 0.327 | 0.334 |
| **Axial** | | | | |
| **Echo** | **1** | | **2** | |
| **Slice** | **I** | **II** | **I** | **II** |
| **Motion 1** | 0.395 | 0.553 | 0.223 | 0.159 |
| **Motion 2** | 5.279 | 5.555 | 11.693 | 6.487 |
| **Motion 3** | 0.179 | 0.160 | 0.261 | 0.116 |

**Table S7:** Artifact power for the motion tolerance experiments performed with the δMB-UTE sequence.

| **Sagittal** | | | | | | | | |
| --- | --- | --- | --- | --- | --- | --- | --- | --- |
| **Echo** | **1** | | | | **2** | | | |
| **Slice** | **I** | **II** | **III** | **IV** | **I** | **II** | **III** | **IV** |
| **Motion 1** | 0.085 | 0.104 | 0.107 | 0.045 | 0.098 | 0.128 | 0.140 | 0.078 |
| **Motion 2** | 0.741 | 0.704 | 1.106 | 0.277 | 2.328 | 1.171 | 1.699 | 1.975 |
| **Motion 3** | 0.090 | 0.089 | 0.078 | 0.049 | 0.139 | 0.088 | 0.102 | 0.115 |
| **Axial** | | | | | | | | |
| **Echo** | **1** | | | | **2** | | | |
| **Slice** | **I** | **II** | **III** | **IV** | **I** | **II** | **III** | **IV** |
| **Motion 1** | 0.125 | 0.095 | 0.129 | 0.057 | 0.163 | 0.119 | 0.085 | 0.055 |
| **Motion 2** | 1.303 | 1.072 | 1.927 | 0.918 | 3.262 | 2.166 | 1.890 | 1.206 |
| **Motion 3** | 0.183 | 0.209 | 0.294 | 0.081 | 0.224 | 0.118 | 0.113 | 0.088 |

**Supporting Figures:**

**
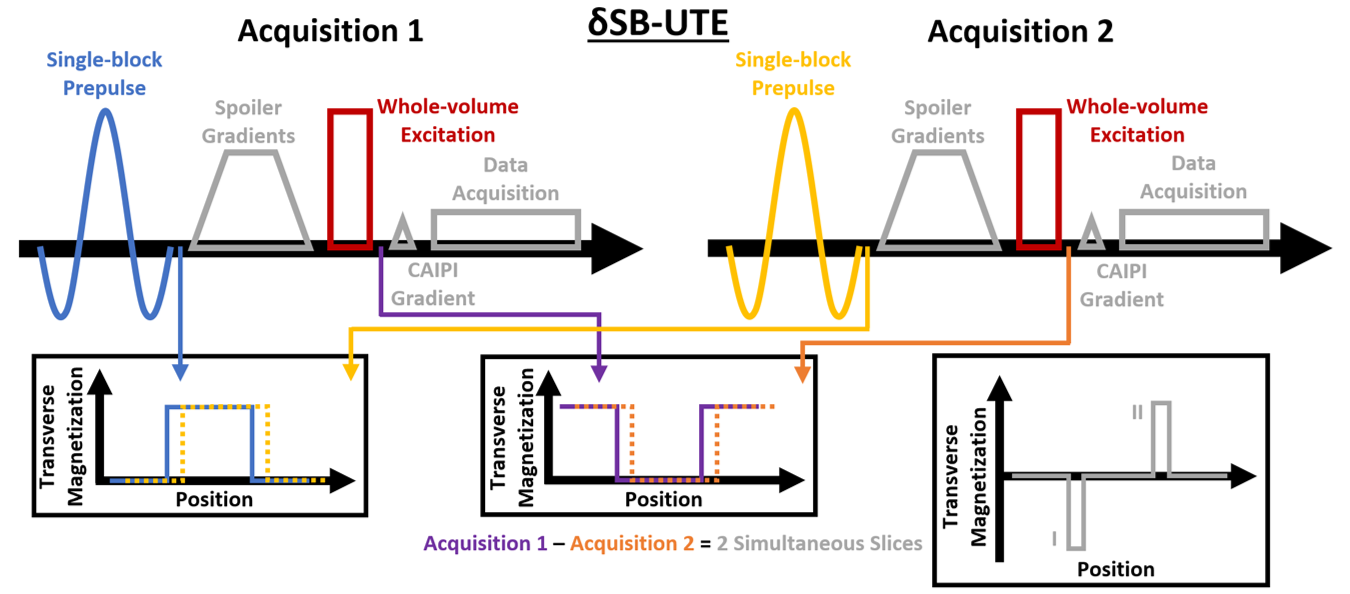
**

**Figure S1: A schematic diagram of the δSB-UTE sequence showing transverse magnetization profiles throughout the sequence and the subtraction of subsequent acquisitions.** In the first acquisition a single-block prepulse (blue) tips a wide region of magnetization into the transverse plane (blue) and spoiler gradients dephase the transverse magnetization. A non-selective rectangular pulse (red) excites the surrounding magnetization (purple) and data acquisition begins following the CAIPI gradient and transmit/receive switching. In the second acquisition, frequency modulation of the prepulse (yellow) shifts the saturation blocks (yellow, orange) along the slice-select direction. Subtraction of subsequent acquisitions enables the reconstruction of two (I and II) simultaneous slices.

**
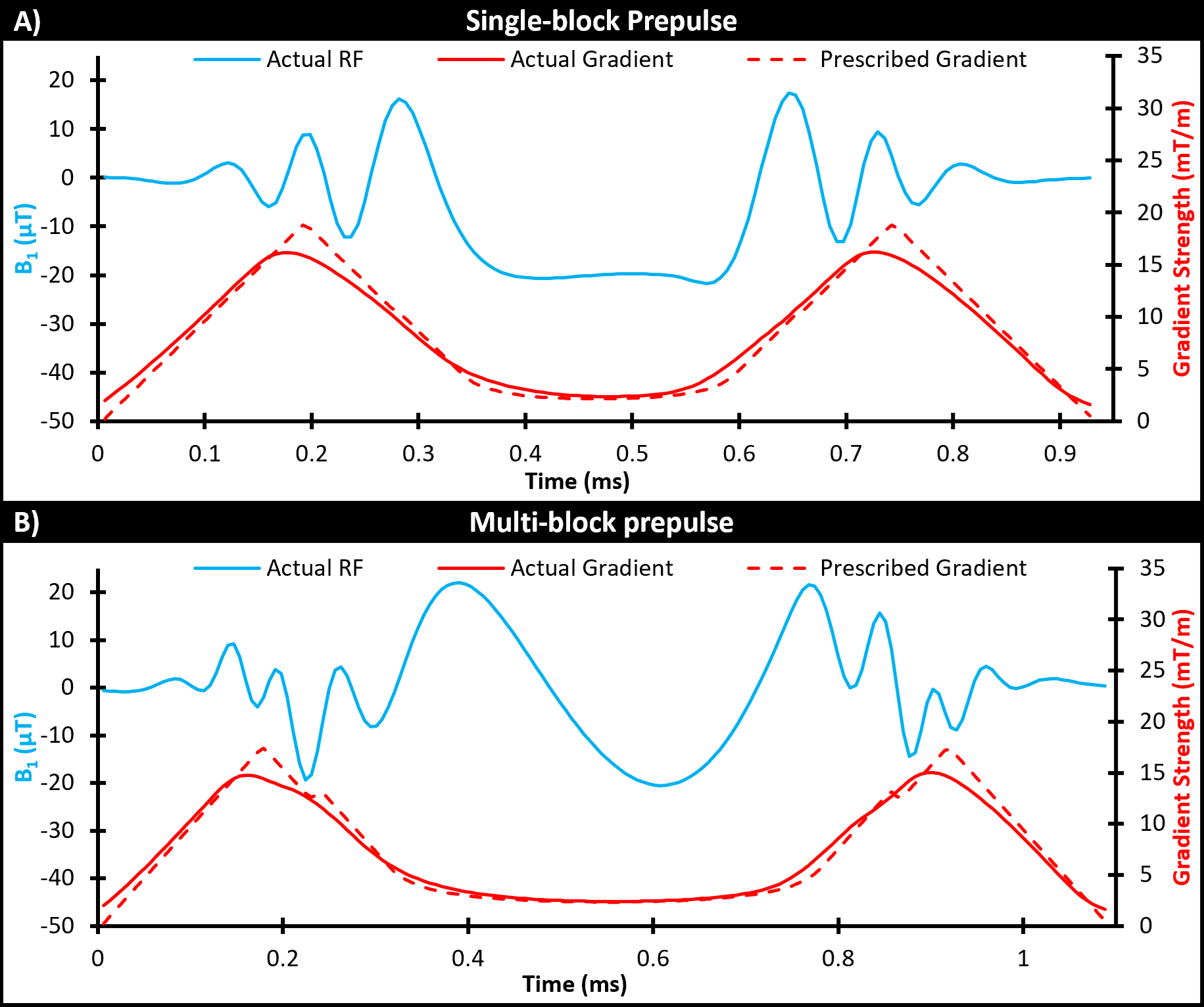
**

**Figure S2:** **The RF waveform (blue), prescribed gradient (red, dashed), and expected gradient (red, solid) of 70º single-block (A) and multi-block (B) prepulses.** The single-block prepulse has a TBW of 13.2 and saturation block thickness of 40 mm, while the multi-block prepulse has a TBW of 6.6 and saturation block thickness of 20 mm.

**
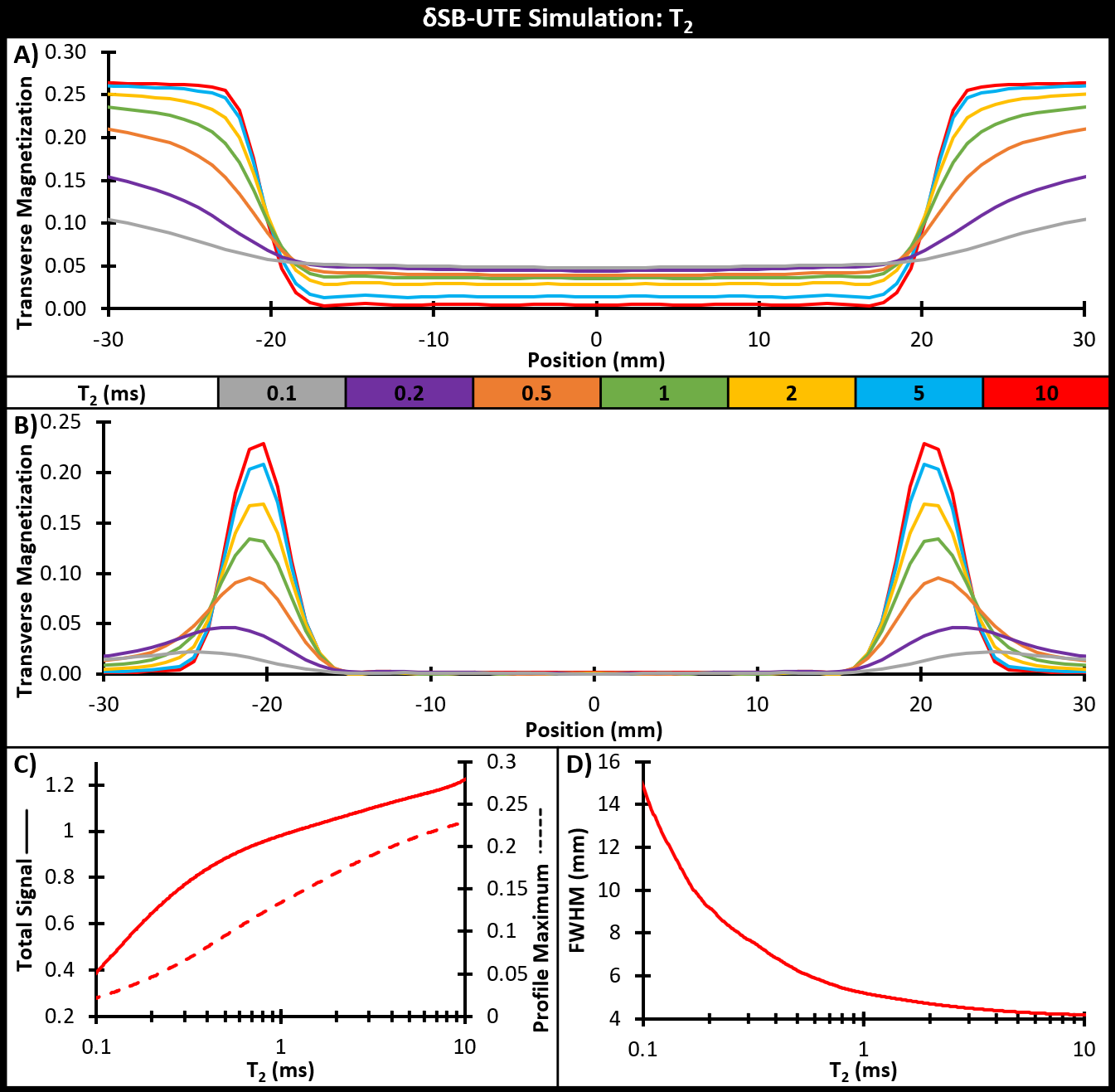
**

**Figure S3: Slice profiles of δSB-UTE sequence simulated with T_2_ = 0.1-10 ms.** Parameters included a prepulse flip angle of 70º, prepulse TBW of 13.2, saturation block thickness of 40 mm, TR of 35 ms, excitation flip angle of 29.9º, and T_1_ of 245 ms. The saturation block was not shifted to produce saturation block profiles (A) and was shifted of 4 mm to produce slice profiles (B). The total signal and profile maximum (C) and FWHM (D) are plotted for each slice.

**
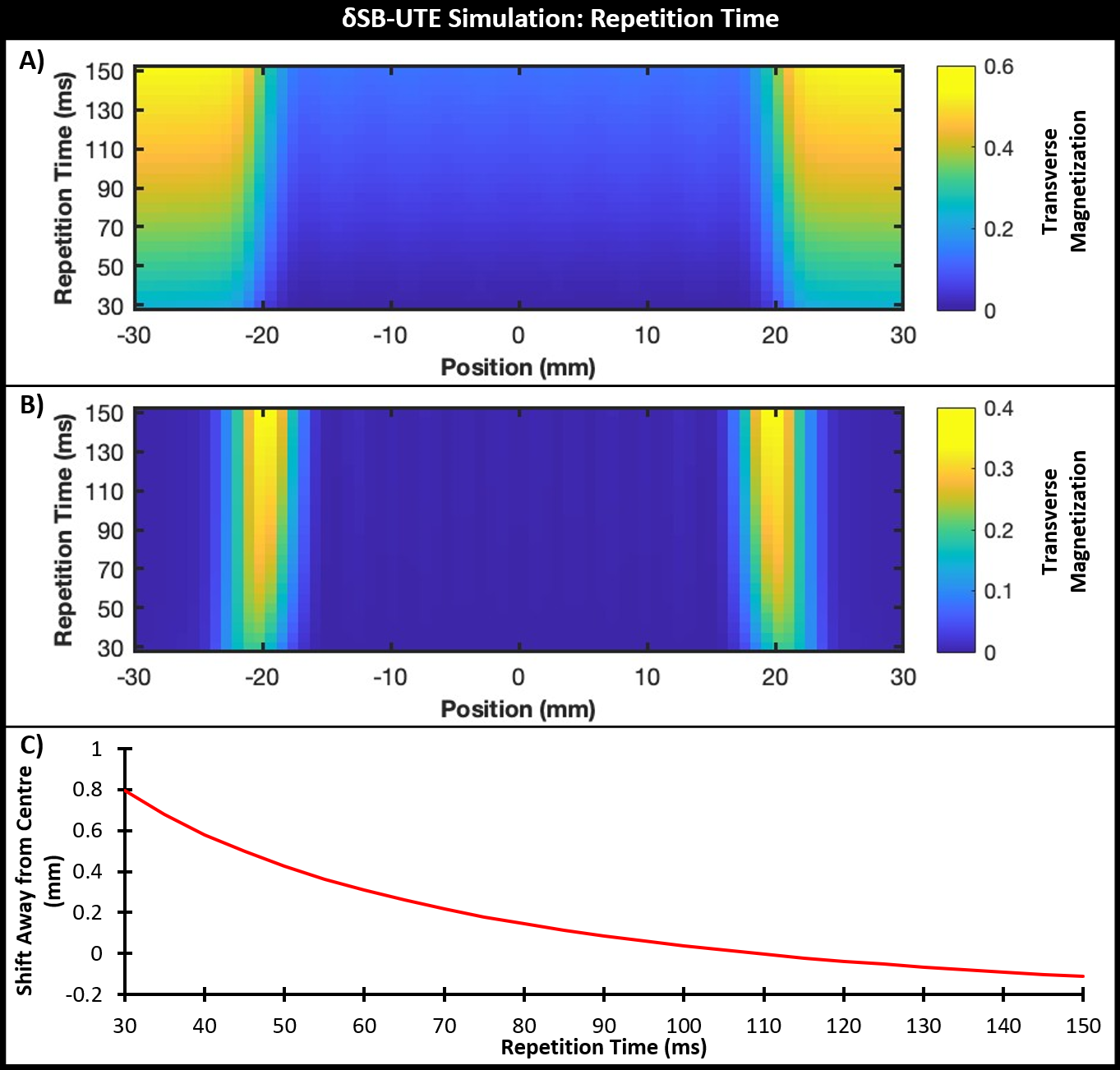
**

**Figure S4: Slice profiles of δSB-UTE sequence simulated with TR = 30-150 ms.** Parameters included a prepulse flip angle of 70º, prepulse TBW of 13.2, saturation block thickness of 40 mm, excitation flip angles of 27.8-57.2º, T_2_ of 5 ms, and T_1_ of 245 ms. The saturation block was not shifted to produce saturation block profiles (A) and was shifted of 4 mm to produce slice profiles (B). The outward shift of the slices away from their expected positions of ±20 mm is plotted (C).

**
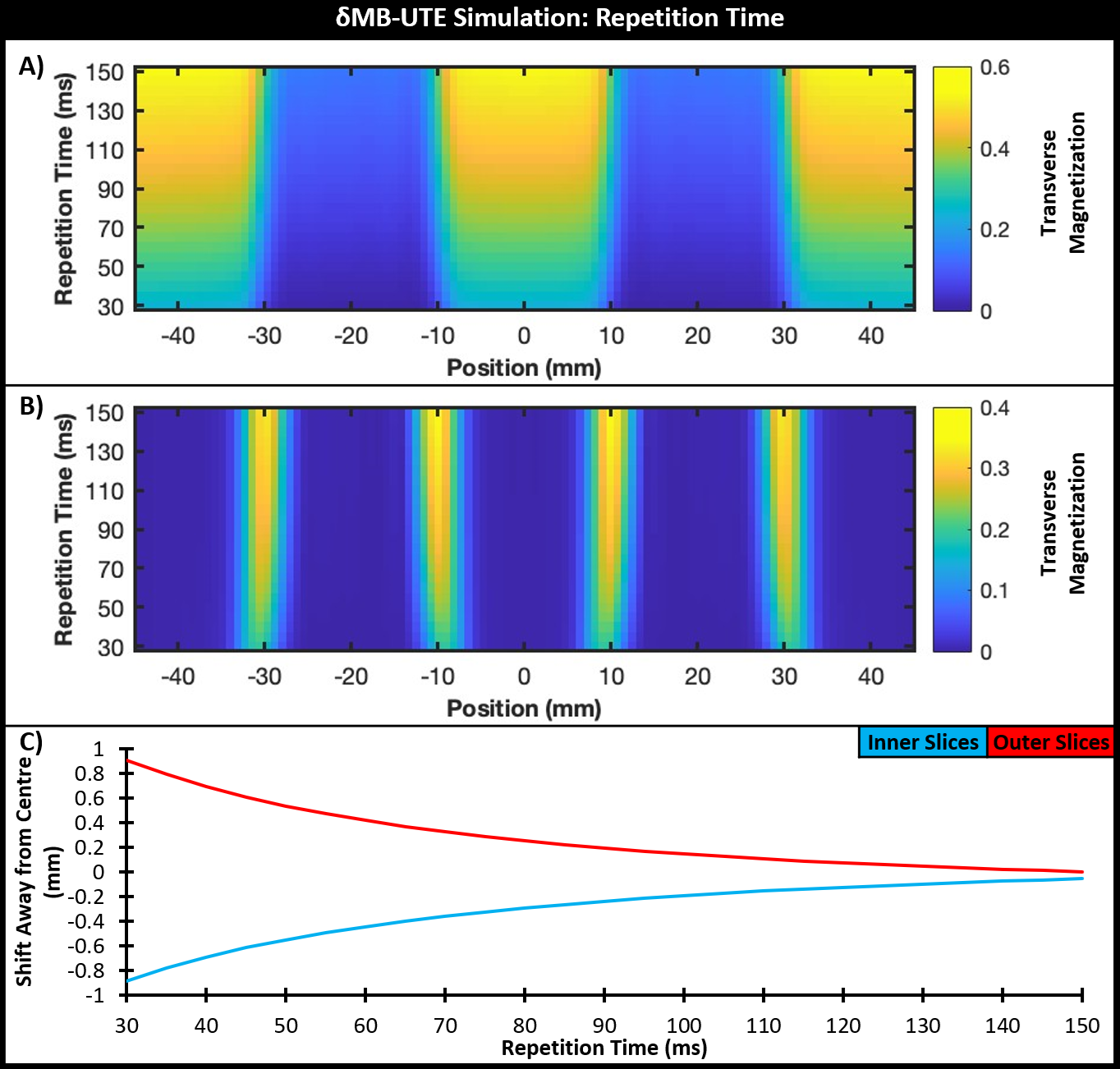
**

**Figure S5: Slice profiles of δMB-UTE sequence simulated with TR = 30-150 ms.** Parameters included a prepulse flip angle of 70º, prepulse TBW of 6.6, saturation block thickness of 20 mm, excitation flip angles of 27.8-57.2º, T_2_ of 5 ms, and T_1_ of 245 ms. The saturation block was not shifted to produce saturation block profiles (A) and was shifted of 4 mm to produce slice profiles (B). The outward shift of the slices away from their expected positions of ±10 mm and ±30 mm is plotted (C).


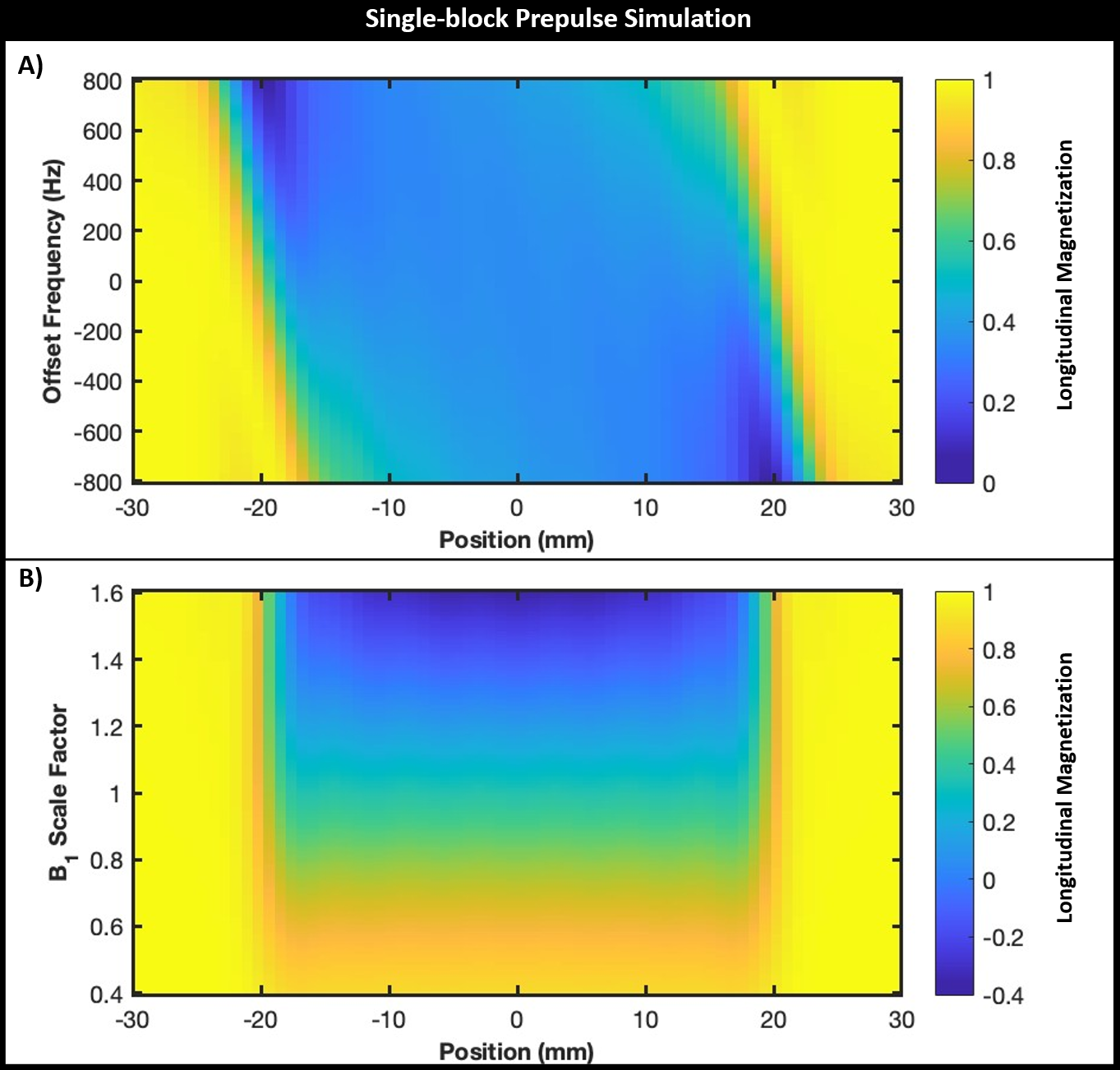


**Figure S6: Simulated longitudinal magnetization profiles after a single iteration of a single-block prepulse for off-resonance frequencies of –800-800 Hz (A) and B_1_ scale factors of 0.4-1.6 (B).** Parameters included a prepulse flip angle of 70º, TBW of 13.2, saturation block thickness of 40 mm, T_2_ of 5 ms, and T_1_ of 245 ms.

**
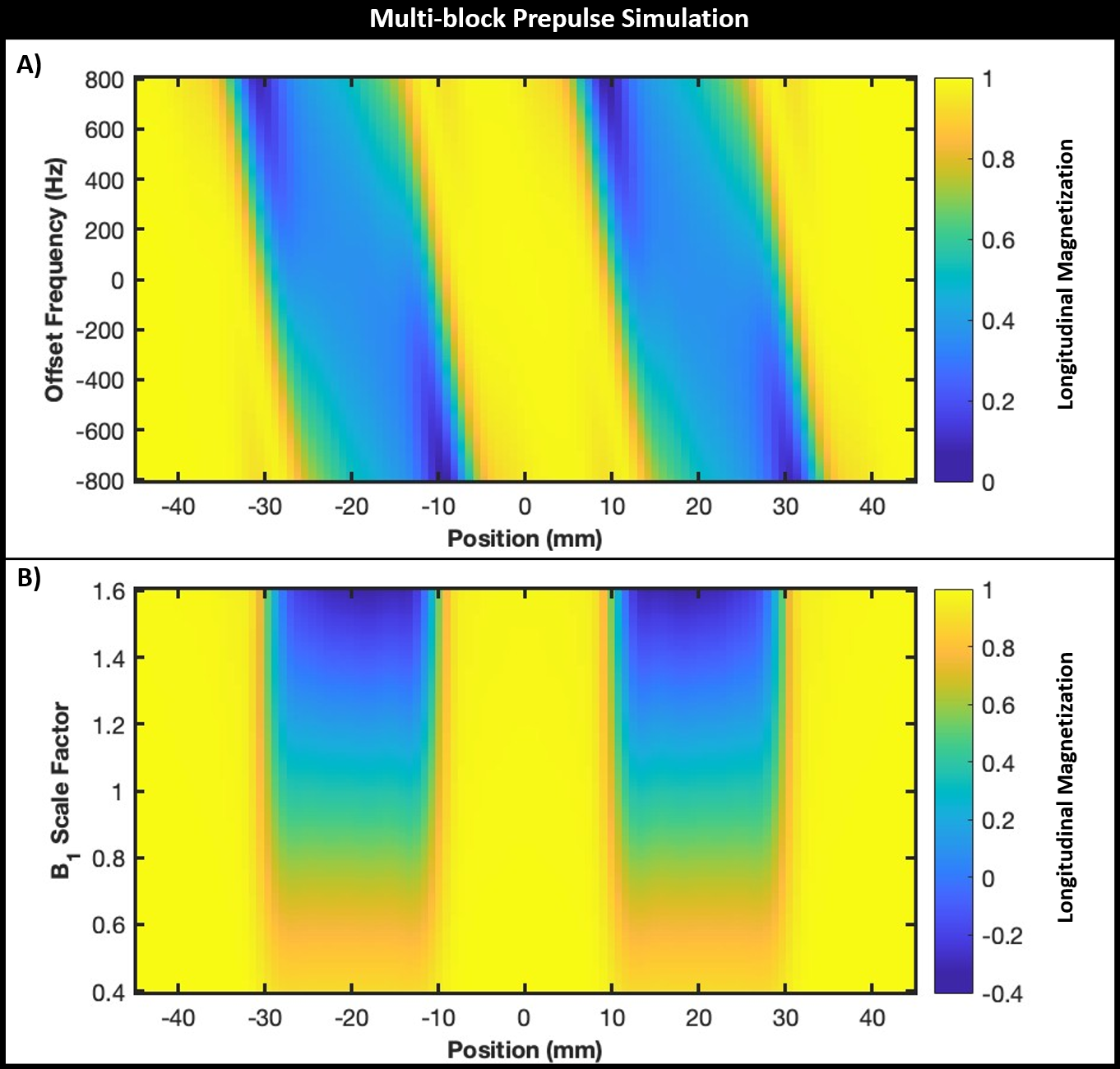
**

**Figure S7: Simulated longitudinal magnetization profiles after a single iteration of a multi-block prepulse for off-resonance frequencies of –800-800 Hz (A) and B_1_ scale factors of 0.4-1.6 (B).** Parameters included a prepulse flip angle of 70º, TBW of 6.6, saturation block thickness of 20 mm, T_2_ of 5 ms, and T_1_ of 245 ms.

**
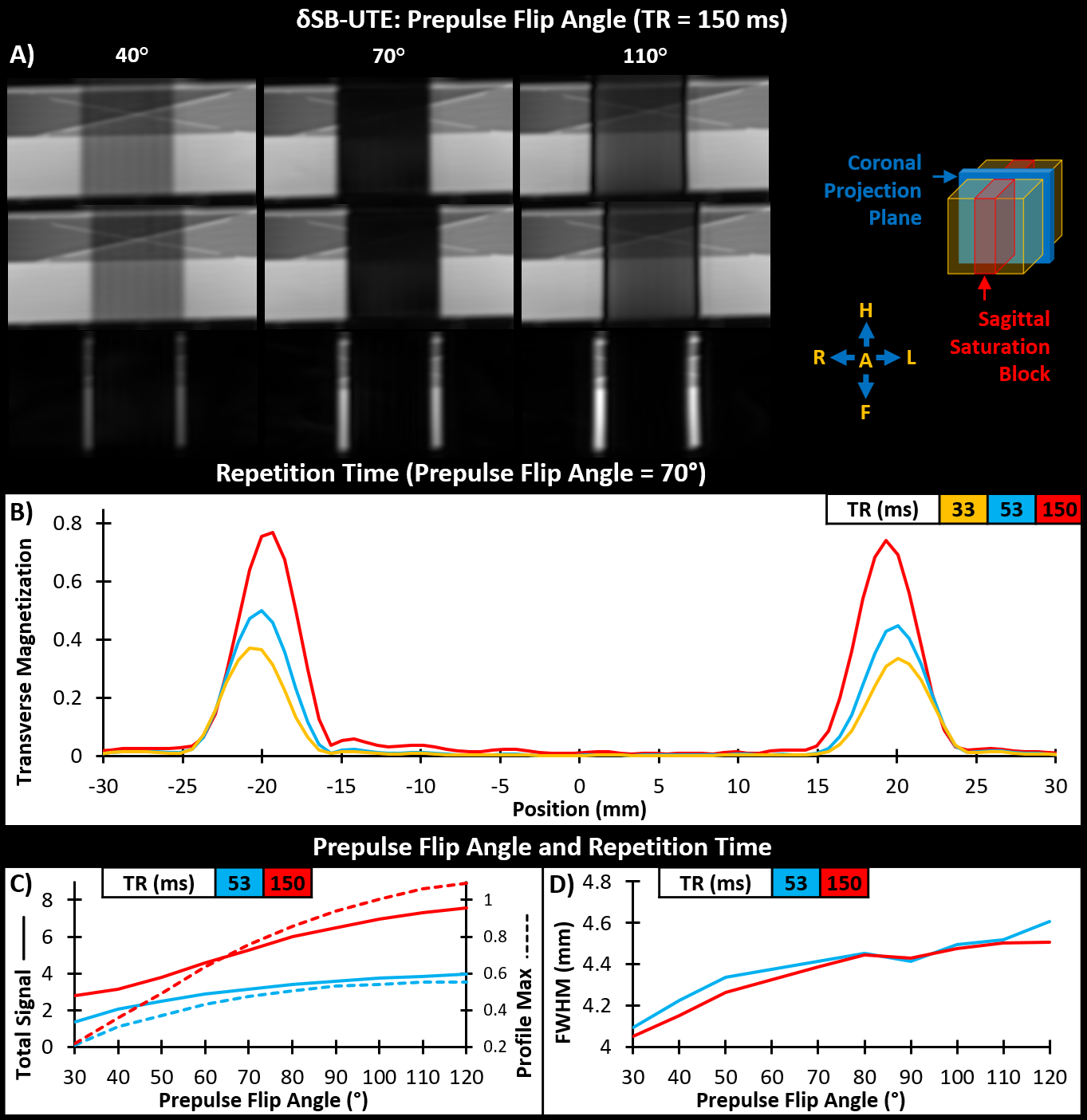
**

**Figure S8. Results of varying prepulse flip angle and TR (phantom experiment 1) for the δSB-UTE sequence.** Saturation block and slice projections obtained with prepulse flip angles of 40, 70, and 110º and a TR of 150 ms and a prepulse flip angle of 70º (A). Slice profiles obtained with TRs of 33, 53, and 150 ms (B). Plots of total signal (solid) and profile maximum (dashed) (C) and FWHM (D) for prepulse flip angles of 30-120º and TRs of 53 and 150 ms. Other parameters included a saturation block thickness of 40 mm, prepulse TBW of 13.2, saturation block shift of 4 mm, and excitation flip angles of 25.7-51.5º.

**
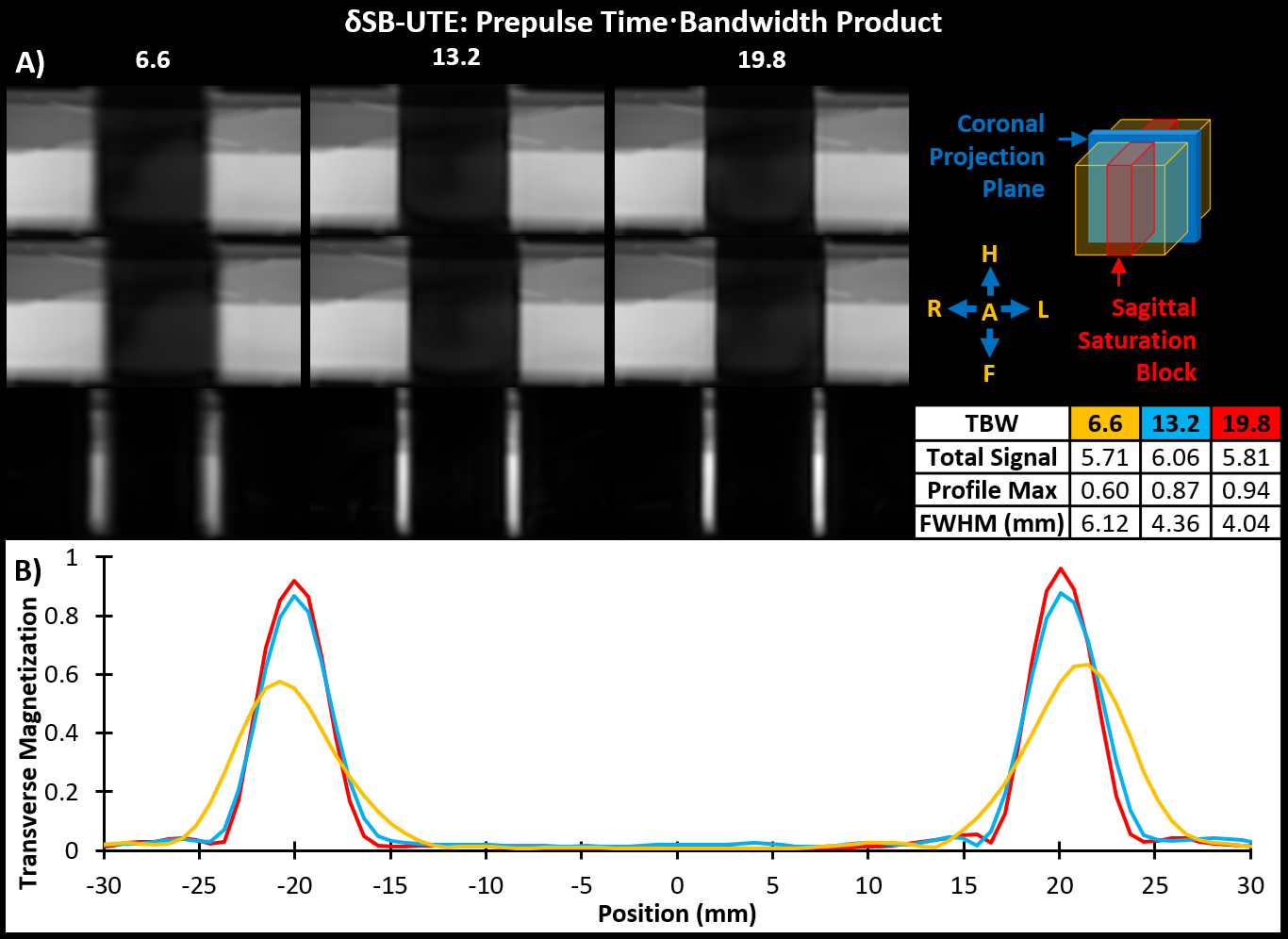
**

**Figure S9. Results of varying prepulse TBW (phantom slice profile experiment 2) for the δSB-UTE sequence.** Saturation block and slice projections (A) and corresponding slice profiles (B) obtained with prepulse TBWs 6.6, 13.2, and 19.8. Total signal, profile maximum, and FWHM are tabulated for each TBW. Other parameters included a prepulse flip angle of 70º, TR of 53 ms, saturation block thickness of 40 mm, saturation block shift of 4 mm, and excitation flip angle of 32.2º.

**
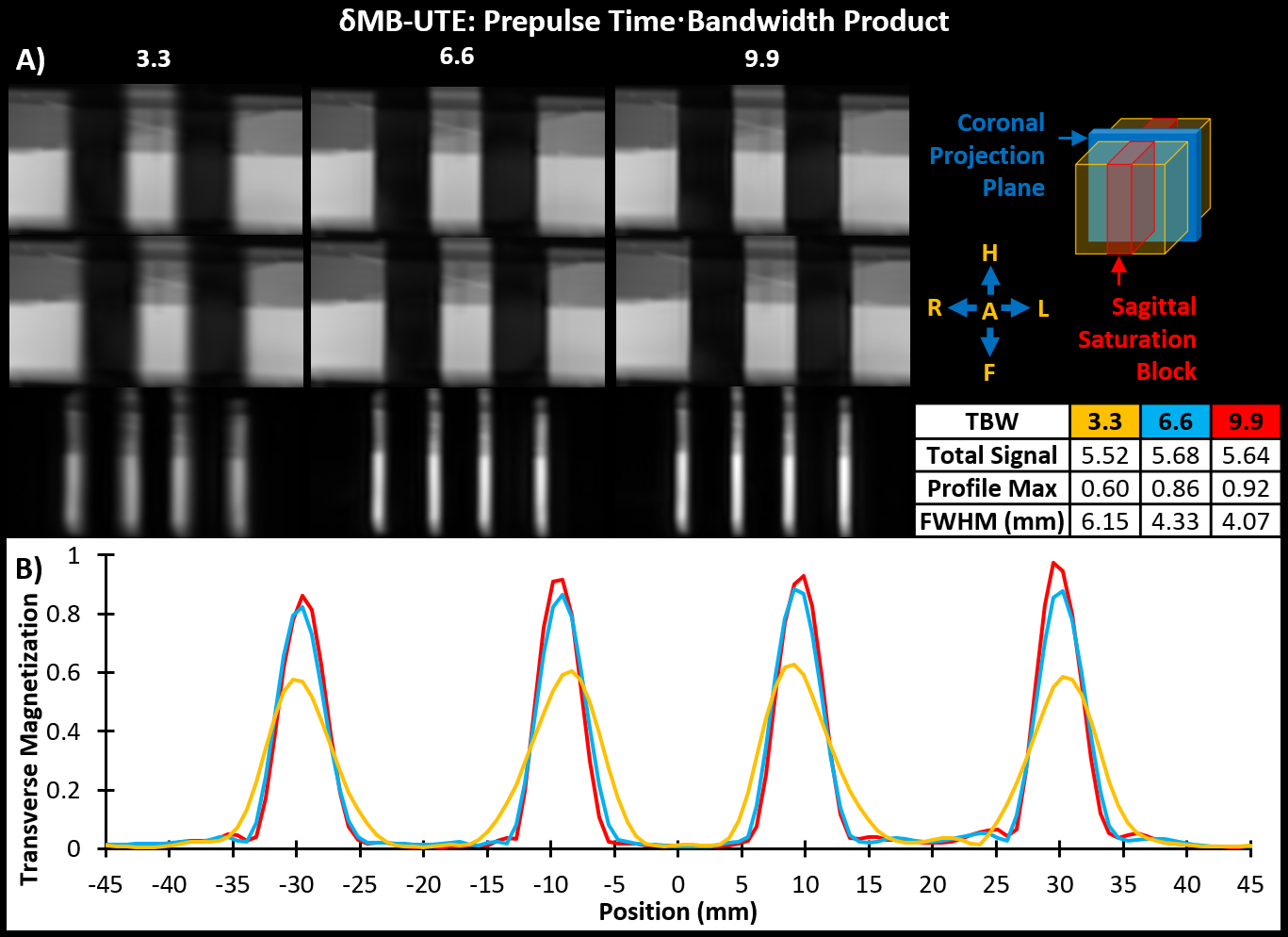
**

**Figure S10. Results of varying prepulse TBW (phantom slice profile experiment 2) for the δMB-UTE sequence.** Saturation block and slice projections (A) and corresponding slice profiles (B) obtained with prepulse TBWs 3.3, 6.6, and 9.9. Total signal, profile maximum, and FWHM are tabulated for each TBW. Other parameters included a prepulse flip angle of 70º, TR of 53 ms, saturation block thickness of 20 mm, saturation block shift of 4 mm, and excitation flip angle of 32.2º.

**
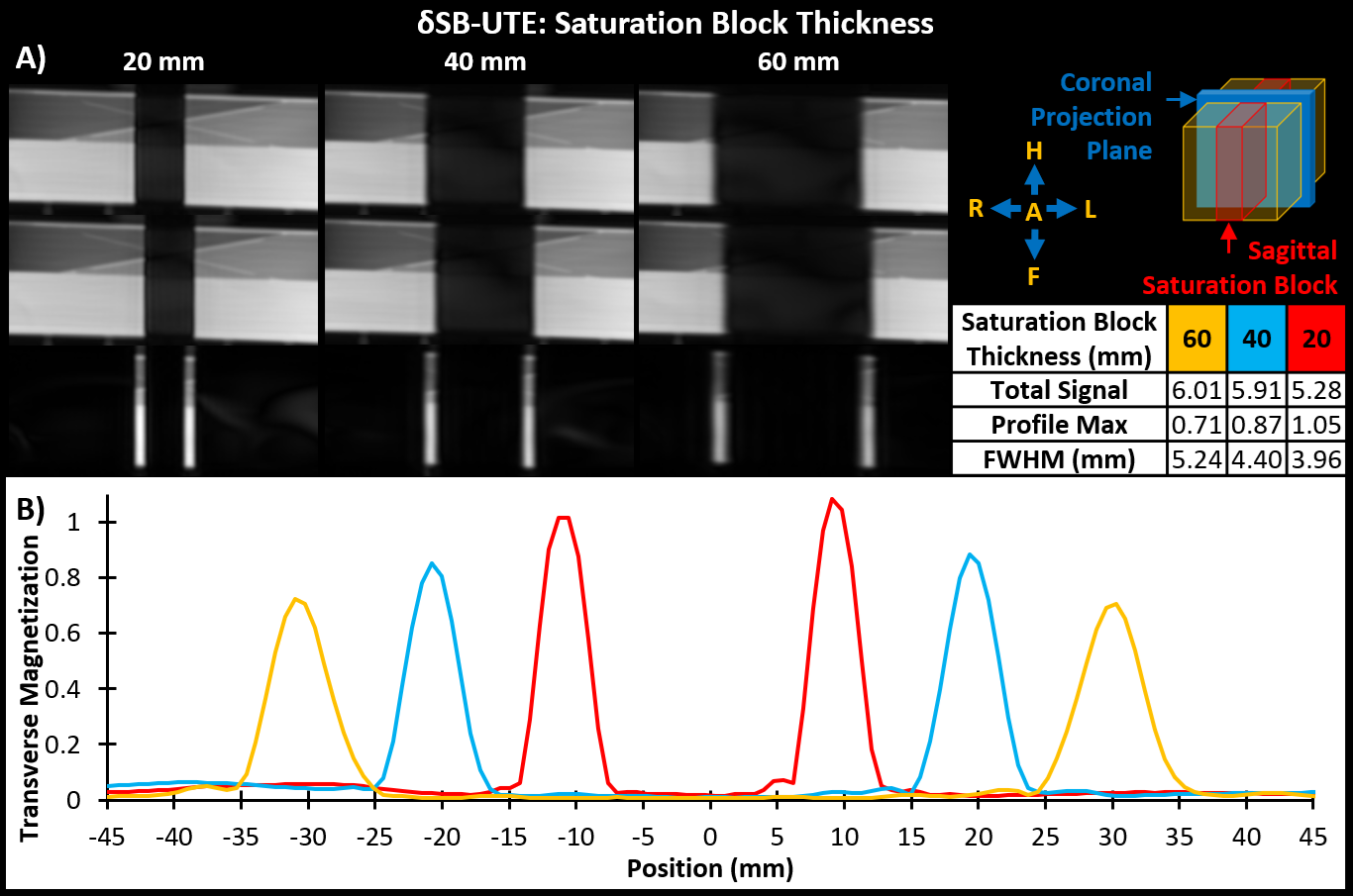
**

**Figure S11. Results of varying saturation block thickness (phantom slice profile experiment 3) for the δSB-UTE sequence.** Saturation block and slice projections (A) and corresponding slice profiles (B) obtained with saturation block thicknesses of 20, 40, and 60 mm. Total signal, profile maximum, and FWHM are tabulated for each thickness. Other parameters included a prepulse flip angle of 70º, prepulse TBW of 13.2, TR of 53 ms, saturation block shift of 4 mm, and excitation flip angle of 32.2º.

**
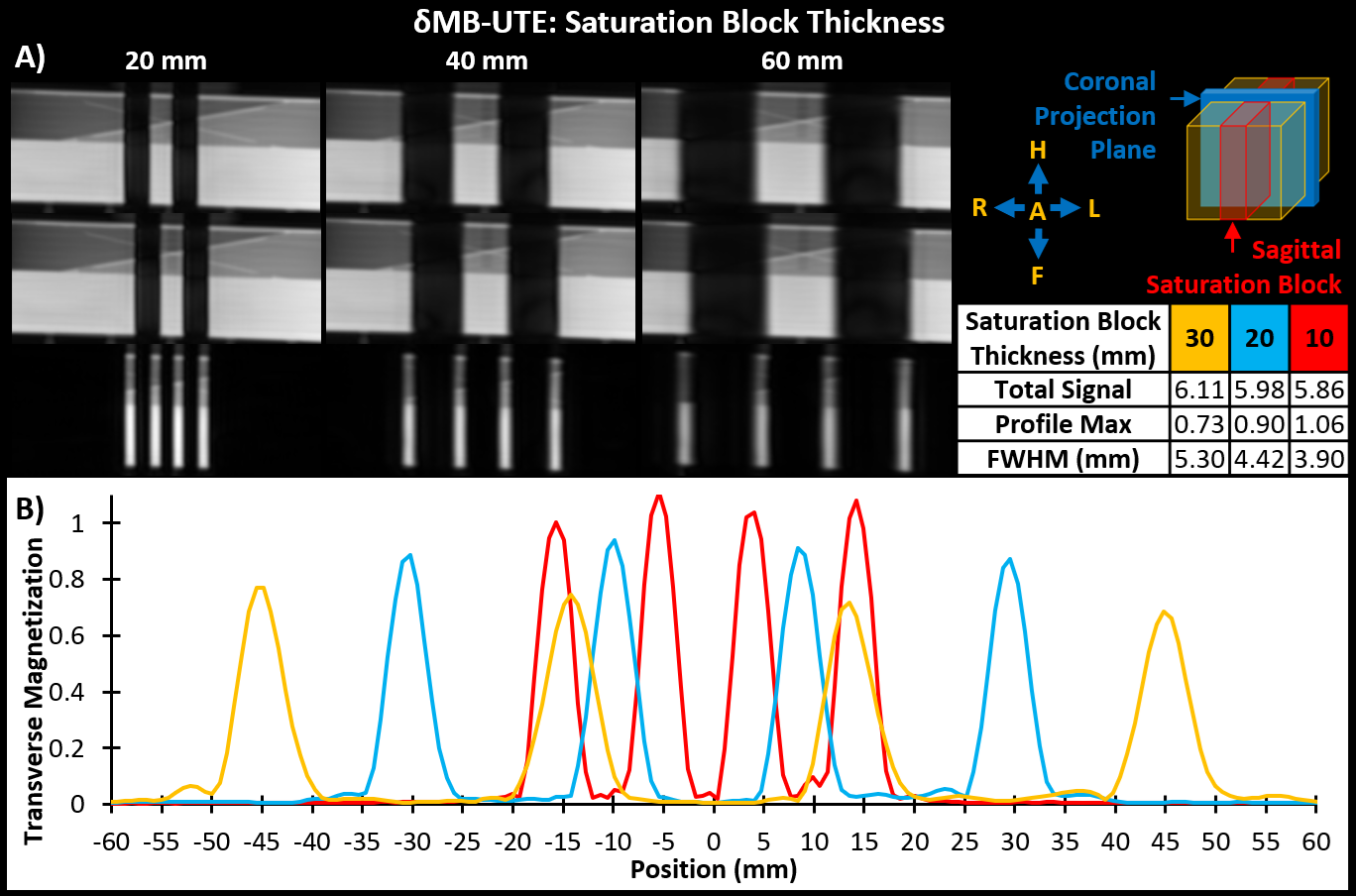
**

**Figure S12. Results of varying saturation block thickness (phantom slice profile experiment 3) for the δMB-UTE sequence.** Saturation block and slice projections (A) and corresponding slice profiles (B) obtained with saturation block thicknesses of 10, 20, and 40 mm. Total signal, profile maximum, and FWHM are tabulated for each thickness. Other parameters included a prepulse flip angle of 70º, prepulse TBW of 6.6, TR of 53 ms, saturation block shift of 4 mm, and excitation flip angle of 32.2º.

**
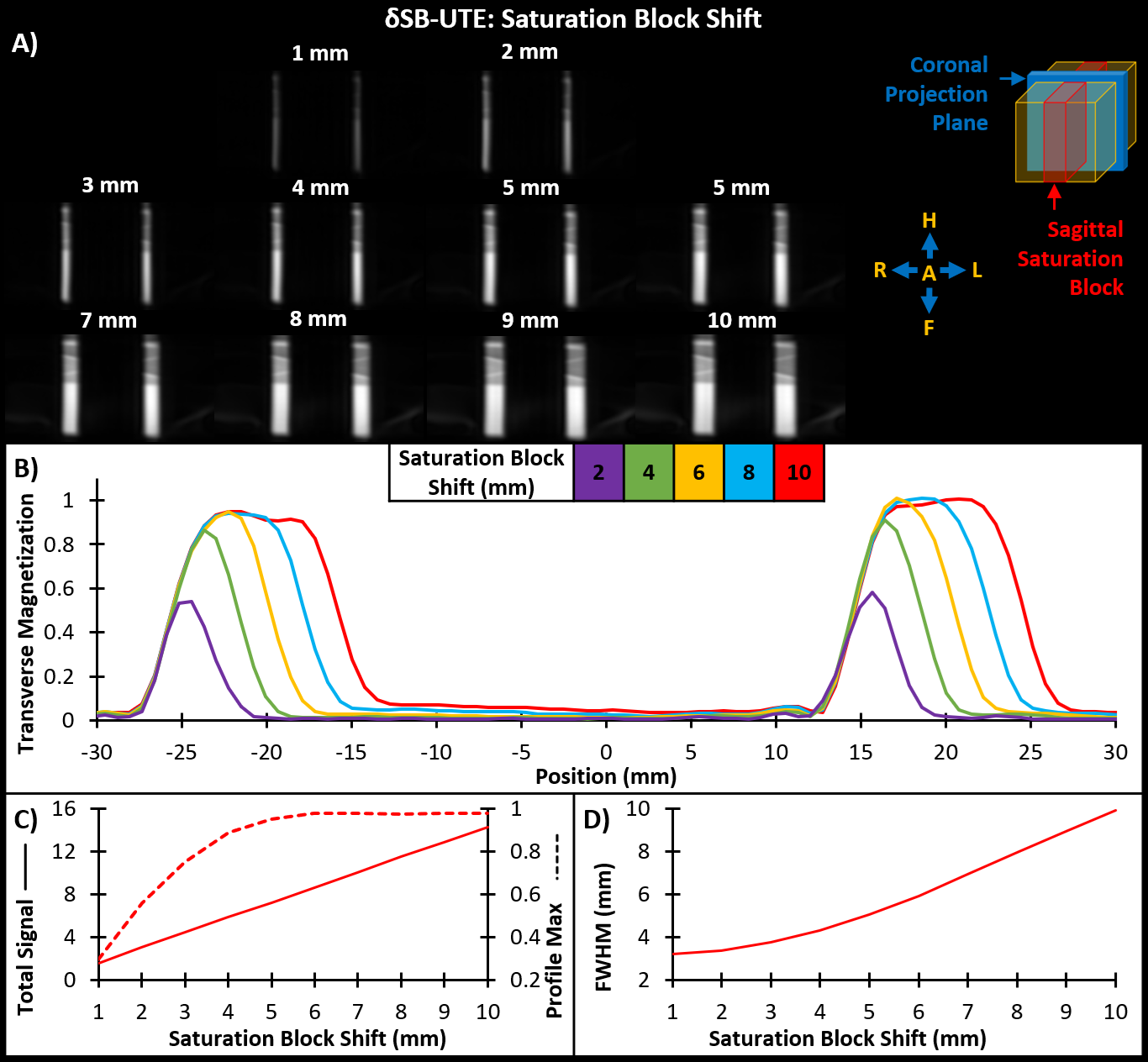
**

**Figure S13. Results of varying saturation block shift (phantom slice profile experiment 4) for the δSB-UTE sequence.** Slice projections (A) and corresponding slice profiles (B) obtained with saturation block shifts of 1-10 mm. Plots of total signal (solid) and profile maximum (dashed) (C) and FWHM (D) for each shift. Other parameters included a prepulse flip angle of 70º, prepulse TBW of 13.2, TR of 53 ms, saturation block thickness of 40 mm, and excitation flip angle of 32.2º.

**
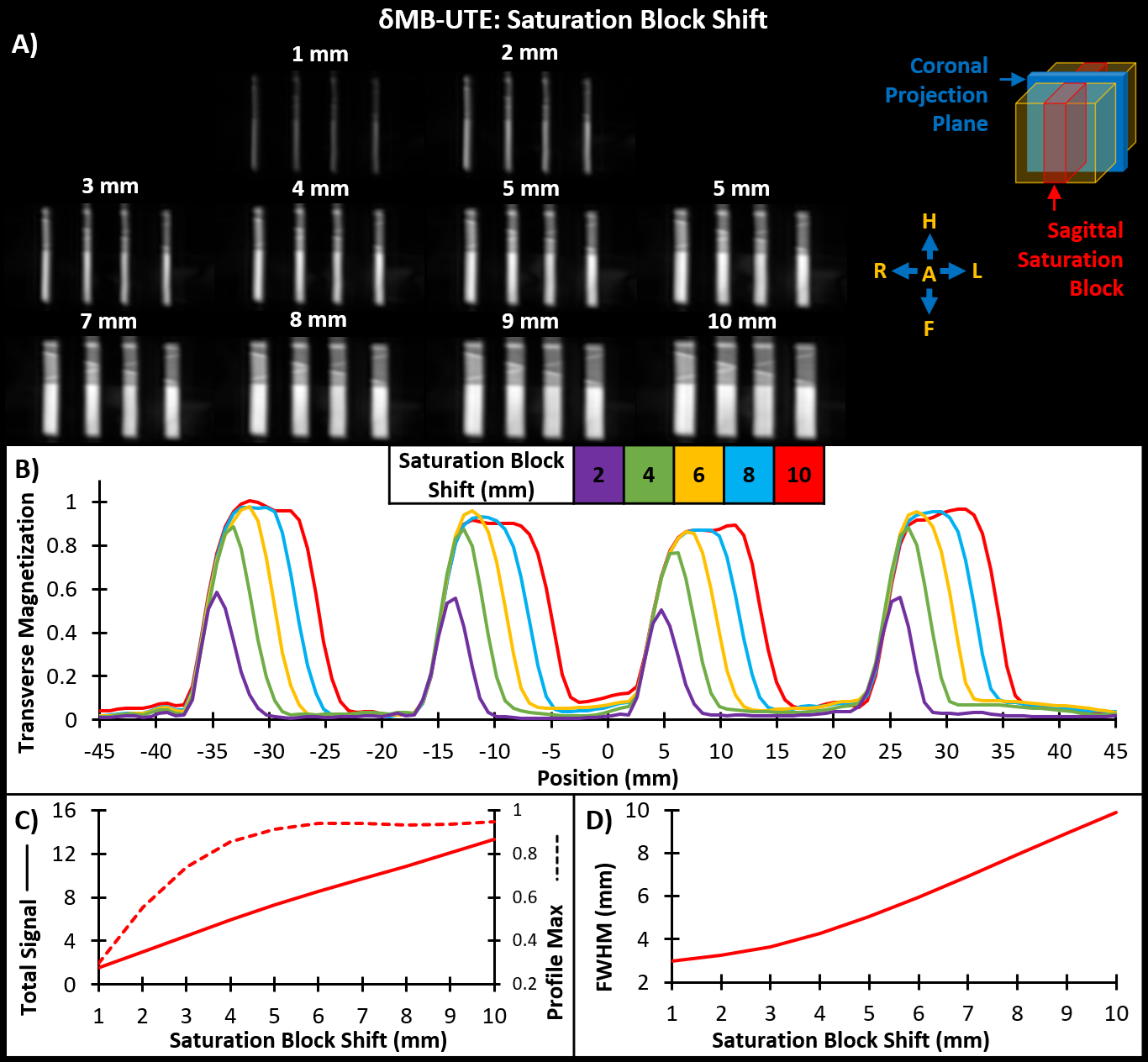
**

**Figure S14. Results of varying saturation block shift (phantom slice profile experiment 4) for the δMB-UTE sequence.** Slice projections (A) and corresponding slice profiles (B) obtained with saturation block shifts of 1-10 mm. Plots of total signal (solid) and profile maximum (dashed) (C) and FWHM (D) for each shift. Other parameters included a prepulse flip angle of 70º, prepulse TBW of 6.6, TR of 53 ms, saturation block thickness of 20 mm, and excitation flip angle of 32.2º.

**
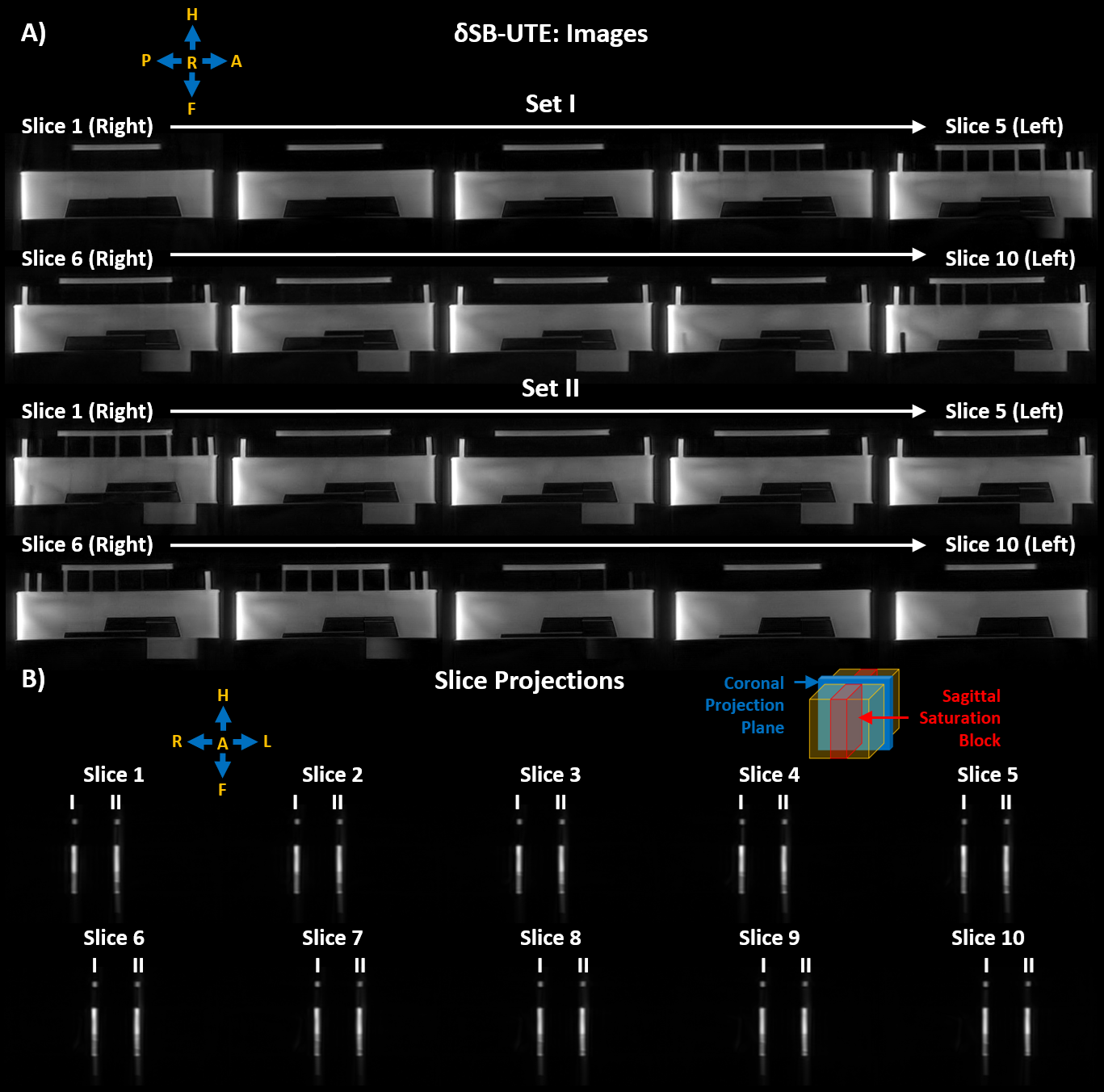
**

**Figure S15. Sagittal images (A) and slice projections (B) obtained with the δSB-UTE sequence over an 80 mm through-plane FOV in a phantom.** The images in sets I and II correspond to the simultaneous slices below. Parameters included a prepulse flip angle of 70º, prepulse TBW of 13.2, saturation block thickness of 40 mm, saturation block shift of 4 mm, TR of 31 ms, excitation flip angle of 24.9º, TE of 0.17 ms, and 11 acquisitions. The scan time for imaging was 2.42 min.

**
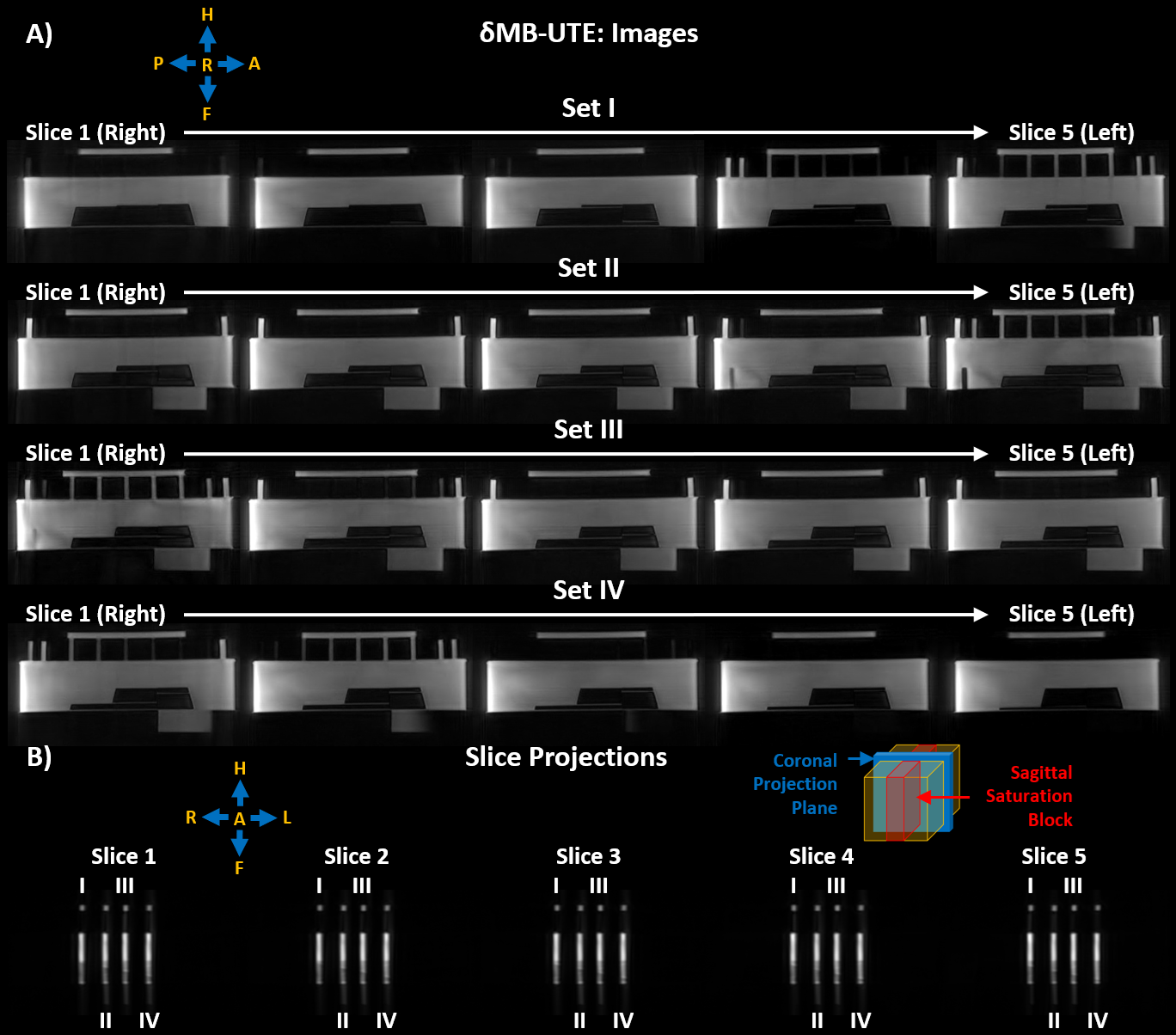
**

**Figure S16. Sagittal images (A) and slice projections (B) obtained with the δMB-UTE sequence over an 80 mm through-plane FOV in a phantom.** The images in sets I, II, III, and IV correspond to the simultaneous slices below. Parameters included a prepulse flip angle of 70º, prepulse TBW of 6.6, saturation block thickness of 20 mm, saturation block shift of 4 mm, TR of 31 ms, excitation flip angle of 24.9º, TE of 0.17 ms, and 6 acquisitions. The scan time for imaging was 1.33 min.

**
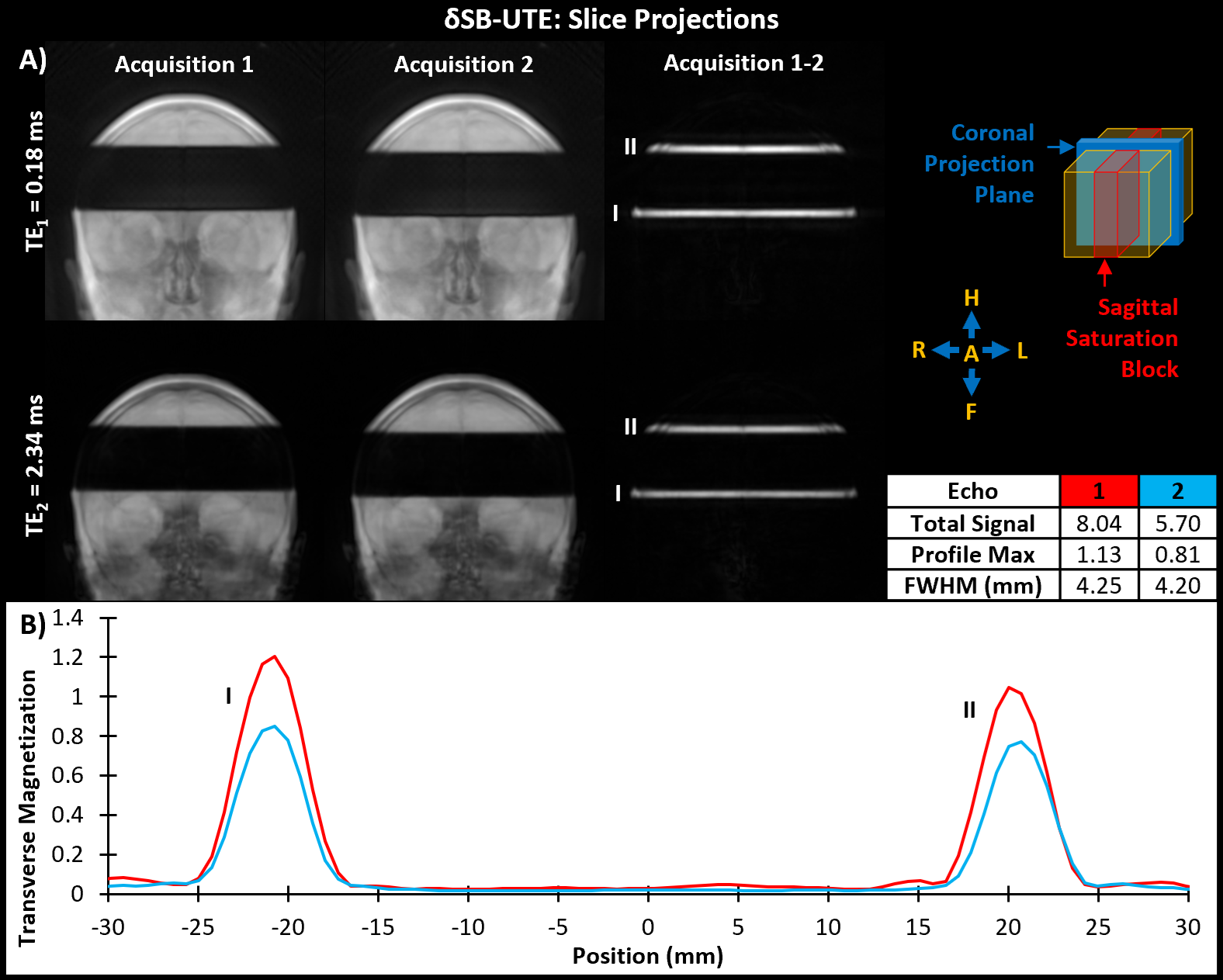
**

**Figure S17. *In-vivo* slice projections (A) and profiles (B) obtained using the δSB-UTE sequence with TE_1_ = 0.18 ms and TE_2_ = 2.34 ms (*in-vivo* experiment 1).** Slices I and II correspond to the axial images in Figure 5A. Parameters included a prepulse flip angle of 70º, prepulse TBW of 13.2, saturation block thickness of 40 mm, saturation block shift of 4 mm, and TR of 34 ms, excitation flip angle of 29.5º.


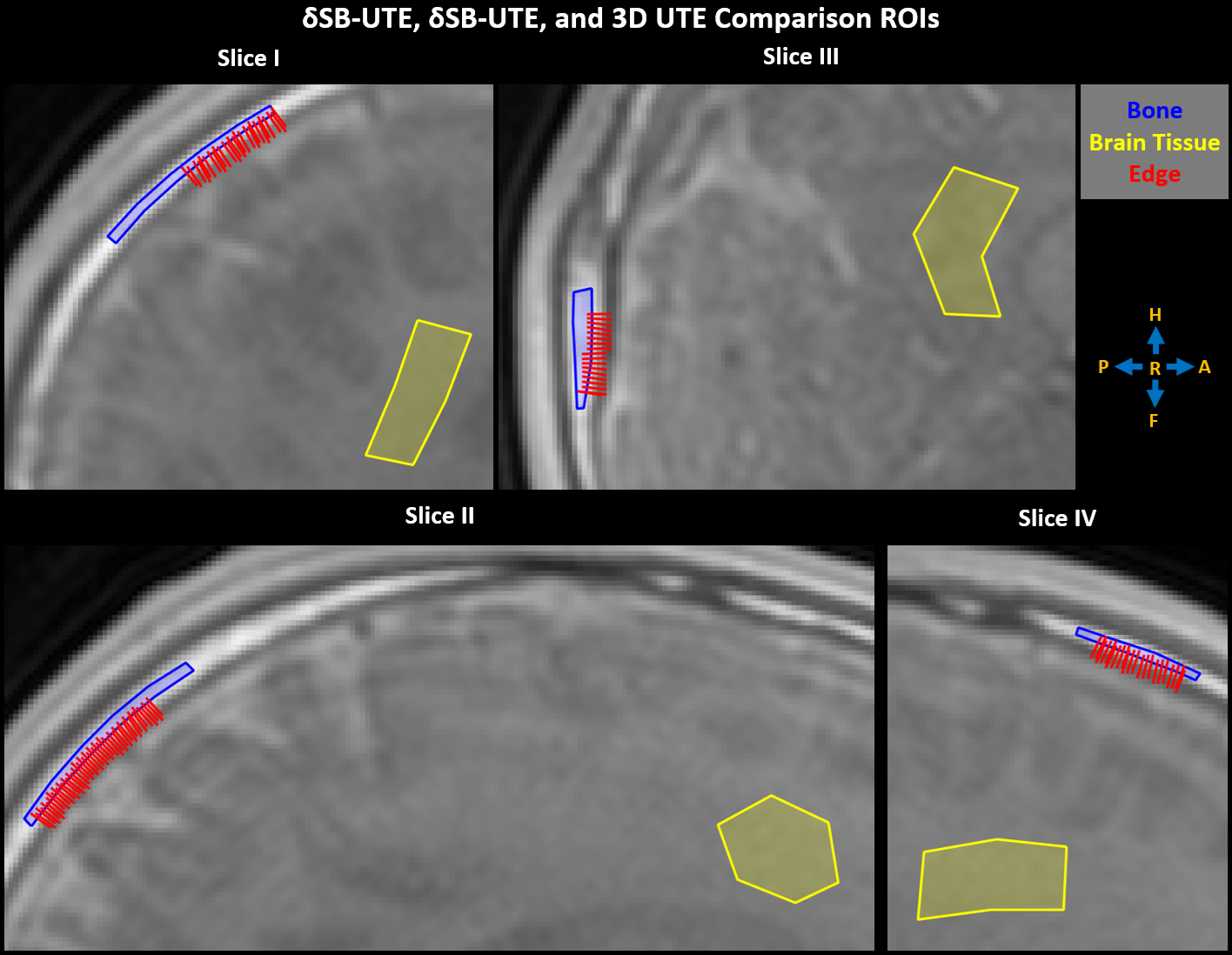


**Figure S18. ROIs used to calculate SNR, CNR, and edge sharpness for images obtained with the δSB-UTE, δMB-UTE, and 3D UTE sequences under matched voxel volumes and scan times (*in-vivo* experiment 3).** The bone (blue), brain tissue (yellow), and edge (red) ROIs are overlaid on zoomed regions of the images obtained with the 3D UTE sequence.


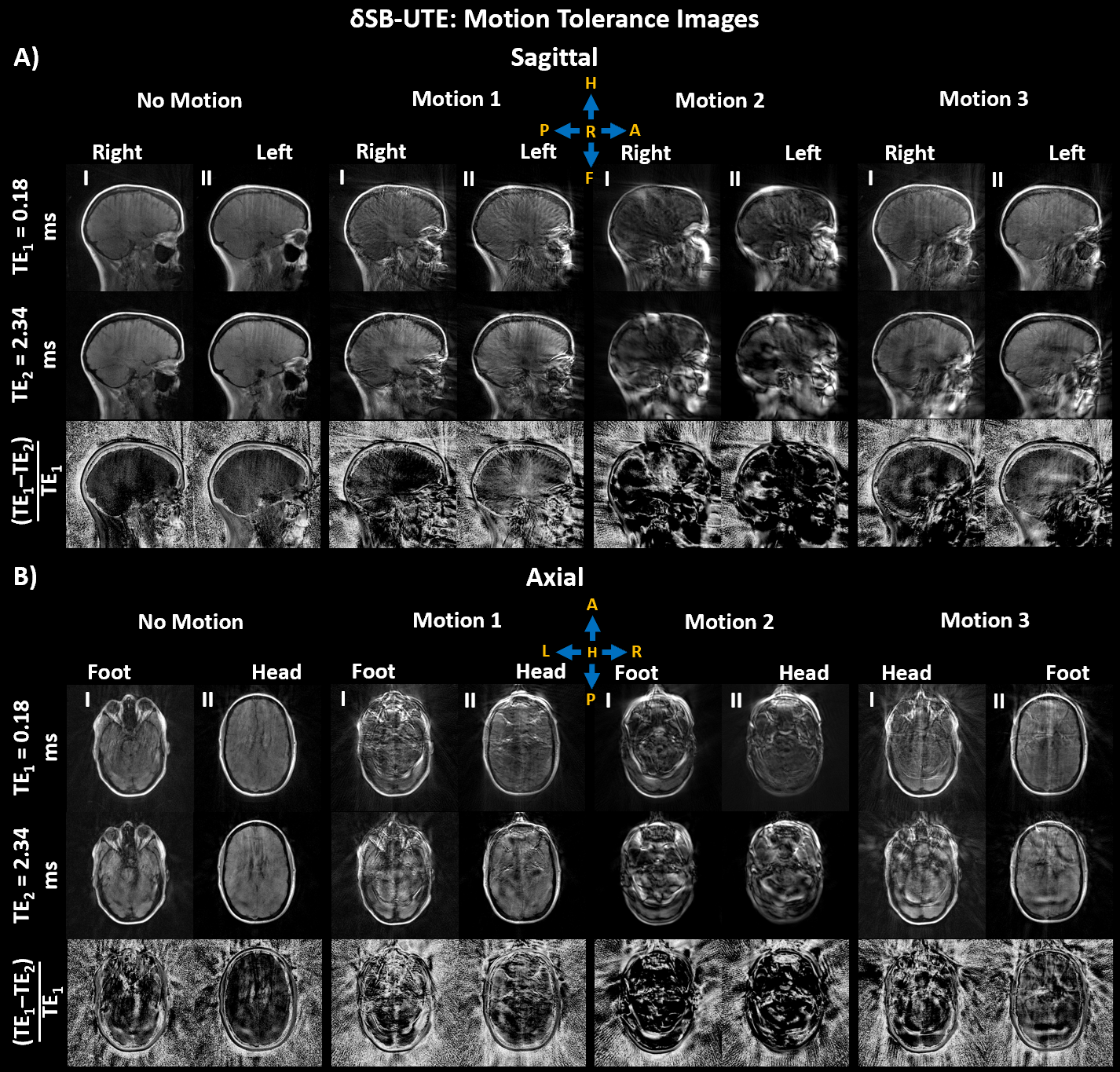


**Figure S19: Results of sagittal (A) and axial (B) *in-vivo* motion tolerance tests performed with the δSB-UTE sequence.** Images obtained without motion, with a few millimeter-pitch nodding (motion 1), a few centimeter-pitch shift in the foot-head direction (motion 2), and with mouth and eye movement (motion 3). Parameters included a prepulse flip angle of 70º, prepulse TBW of 13.2, saturation block thickness of 40 mm, saturation block shift of 4 mm, and TR of 34 ms, excitation flip angle of 29.5º.


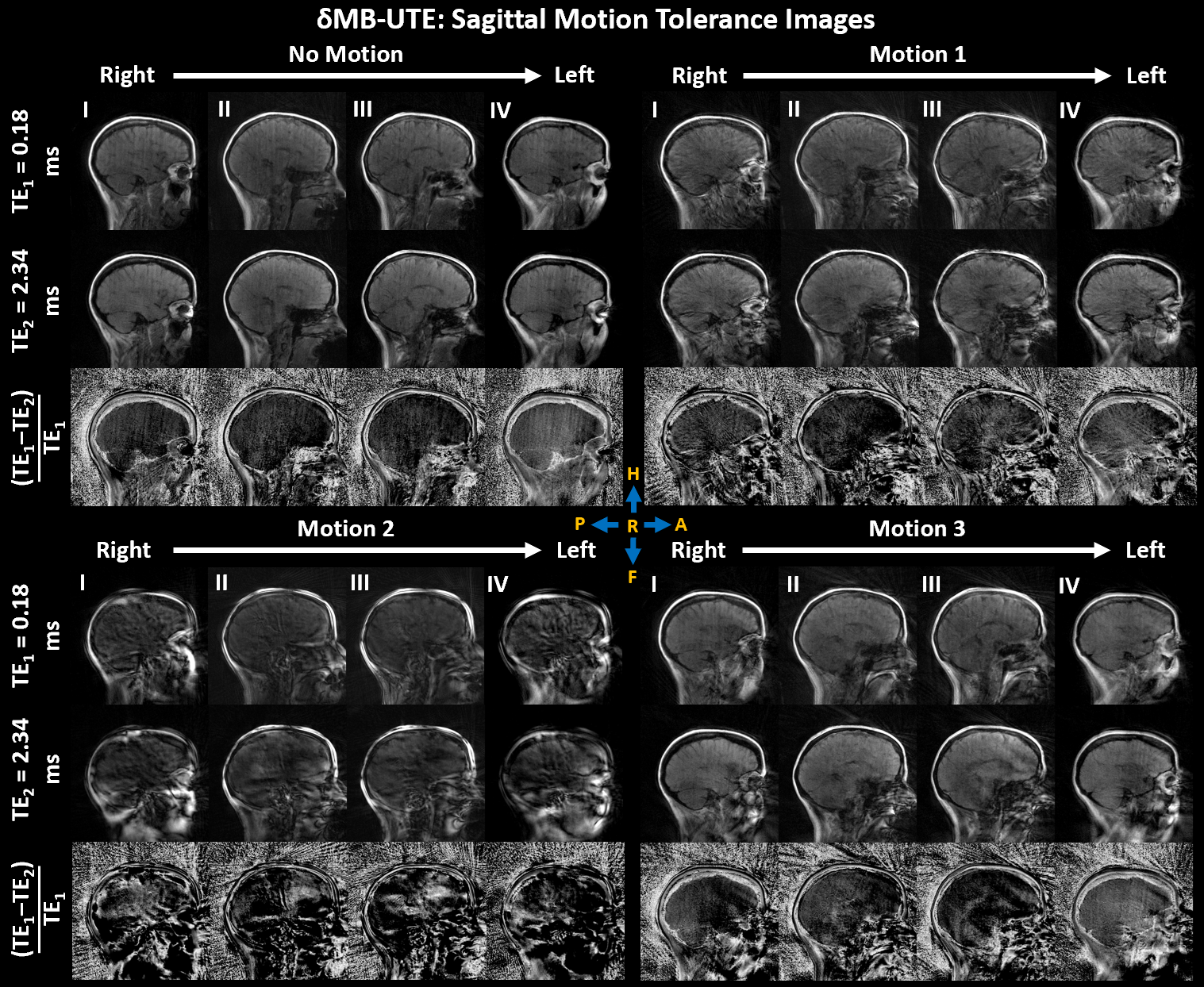


**Figure S20: Results of sagittal *in-vivo* motion tolerance tests performed with the δMB-UTE sequence.** Images obtained without motion, with a few millimeter-pitch nodding (motion 1), a few centimeter-pitch shift in the foot-head direction (motion 2), and with mouth and eye movement (motion 3). Parameters included a prepulse flip angle of 70º, prepulse TBW of 13.2, saturation block thickness of 40 mm, saturation block shift of 4 mm, and TR of 34 ms, excitation flip angle of 29.5º.


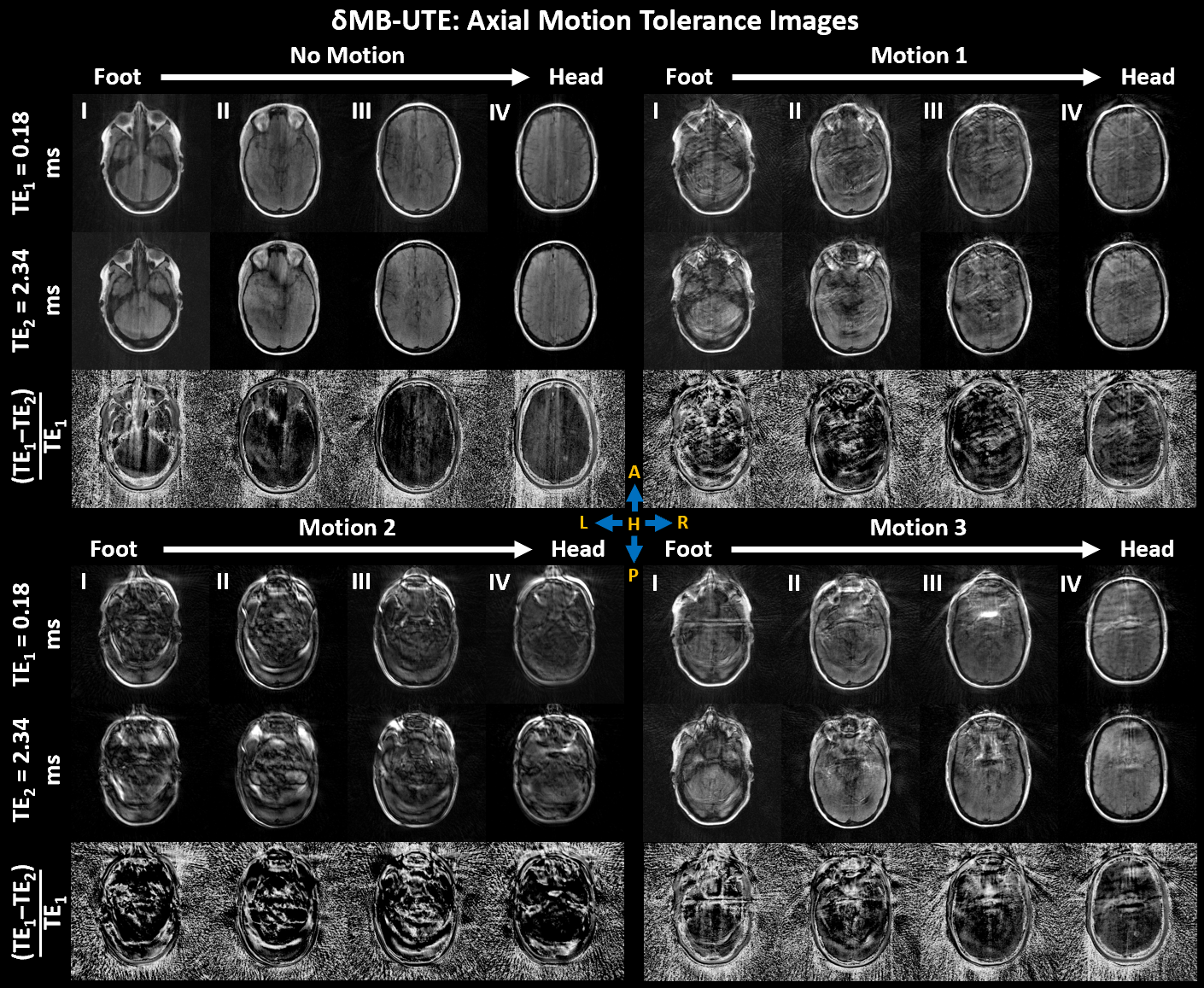


**Figure S21: Results of axial *in-vivo* motion tolerance tests performed with the δMB-UTE sequence.** Images obtained without motion, with a few millimeter-pitch nodding (motion 1), a few centimeter-pitch shift in the foot-head direction (motion 2), and with mouth and eye movement (motion 3). Parameters included a prepulse flip angle of 70º, prepulse TBW of 13.2, saturation block thickness of 40 mm, saturation block shift of 4 mm, and TR of 34 ms, excitation flip angle of 29.5º.


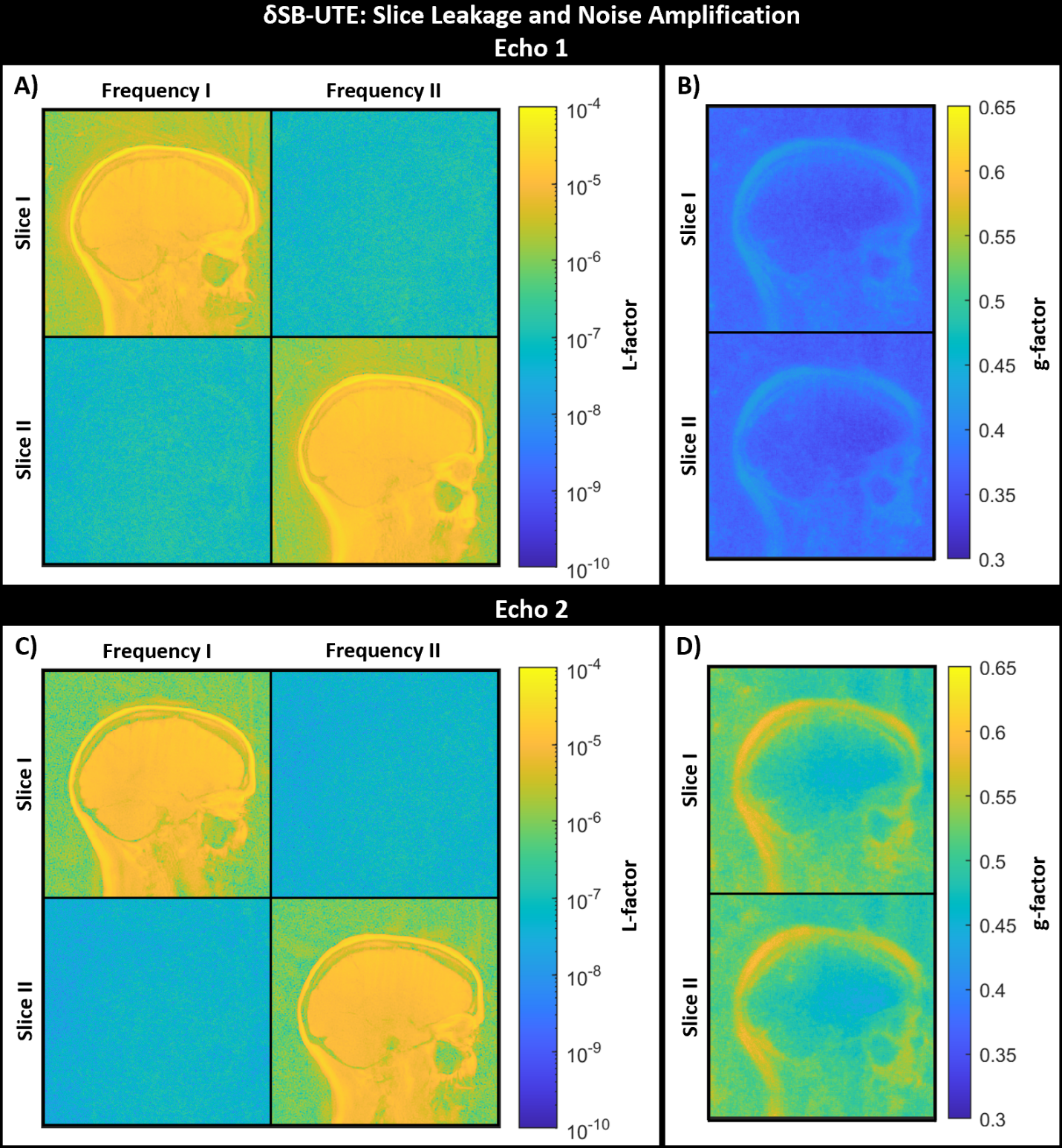


**Figure S22: L-factor and g-factor maps for echo 1 (A and B, respectively) and echo 2 (C and D, respectively) of the δSB-UTE sequence.** The acceleration factor of the acquisition used for the δSB-UTE sequence was 3.1 relative to the fully-sampled reference acquisition.

**
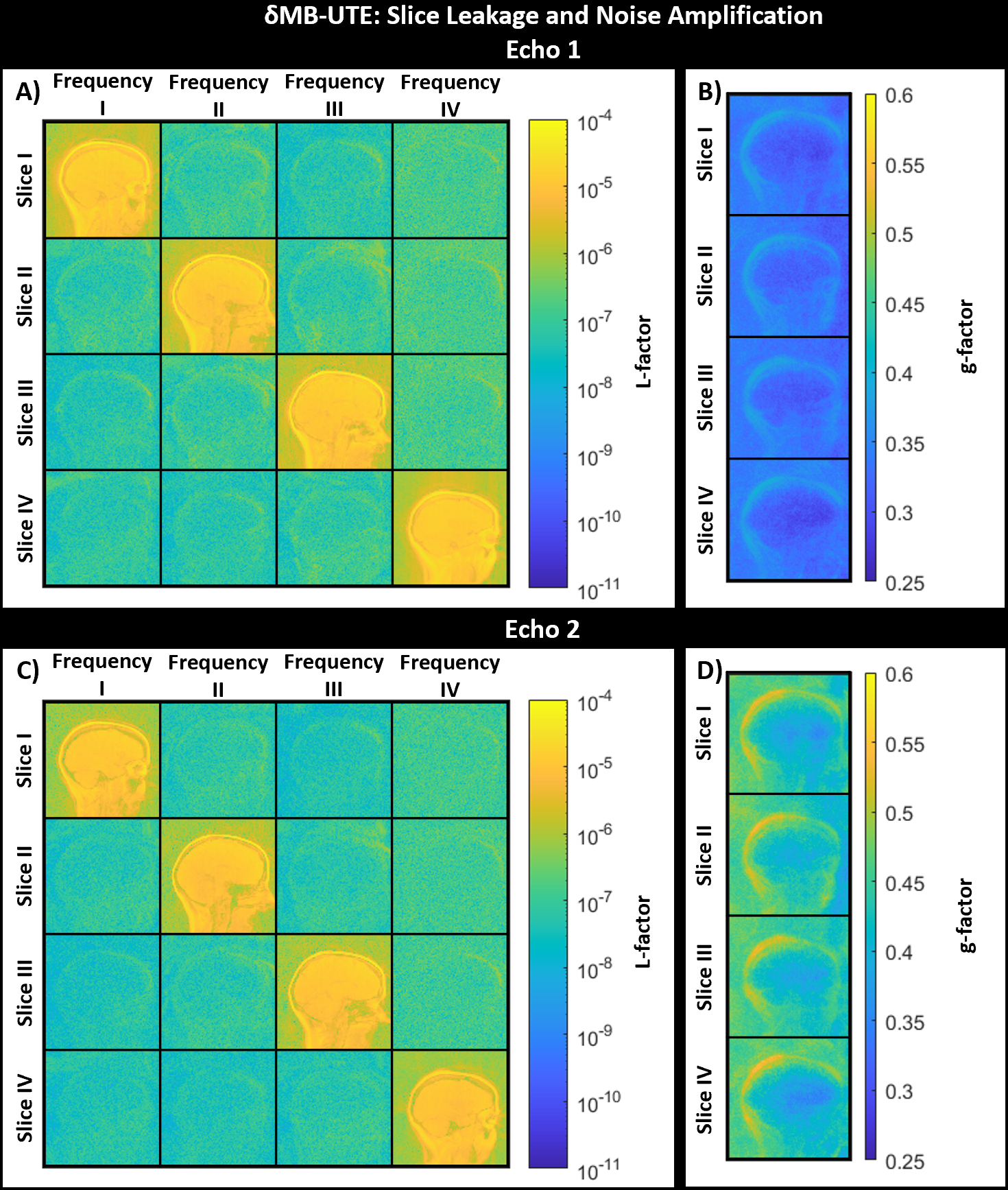
**

**Figure S23: L-factor and g-factor maps for echo 1 (A and B, respectively) and echo 2 (C and D, respectively) of the δMB-UTE sequence.** The acceleration factor of the acquisition used for the δMB-UTE sequence was 6.3 relative to the fully-sampled reference acquisition.
